# Evolution and functional characterization of CAZymes belonging to subfamily 10 of glycoside hydrolase family 5 (GH5_10) in two species of phytophagous beetles

**DOI:** 10.1371/journal.pone.0184305

**Published:** 2017-08-30

**Authors:** André Busch, Grit Kunert, David G. Heckel, Yannick Pauchet

**Affiliations:** 1 Entomology, Max Planck Institute for Chemical Ecology, Jena, Germany; 2 Biochemistry, Max Planck Institute for Chemical Ecology, Jena, Germany; Institut National de la Recherche Agronomique, FRANCE

## Abstract

Hemicelluloses, such as xyloglucan, xylan and mannans, consist of a heterogeneous array of plant-derived polysaccharides that form the plant cell wall. These polysaccharides differ from each other in their structure and physiochemical properties, but they share a β-(1,4)-linked sugar backbone. Hemicelluloses can be hydrolyzed by plant-cell-wall-degrading enzymes (PCWDEs), which are widely distributed in phytopathogenic microbes. Recently, it has become apparent that phytophagous beetles also produce their own PCWDEs. Our previous work identified genes encoding putative mannanases belonging to the subfamily 10 of glycoside hydrolase (GH) family 5 (GH5_10) in the genomes of the leaf beetle, *Gastrophysa viridula* (Chrysomelidae, Chrysomelinae; one gene), and of the bean beetle, *Callosobruchus maculatus* (Chrysomelidae, Bruchinae; four genes). In contrast to proteins from other GH5 subfamilies, GH5_10 proteins are patchily distributed within the tree of life and have so far hardly been investigated. We addressed the following questions: Are beetle-derived GH5_10s active PCWDEs? How did they evolve? What is their physiological function? Using heterologous protein expression and enzymatic assays, we show that the *G*. *viridula* GH5_10 protein is an endo-β-1,4-mannanase. We also demonstrate that only one out of four *C*. *maculatus* GH5_10 proteins is an endo-β-1,4-mannanase, which has additional activity on carboxymethyl cellulose. Unexpectedly, another *C*. *maculatus* GH5_10 protein has evolved to use xylan instead of mannans as a substrate. RNAi experiments in *G*. *viridula* indicate (i) that the sole GH5_10 protein is responsible for breaking down mannans in the gut and (ii) that this breakdown may rather be accessory and may facilitate access to plant cell content, which is rich in nitrogen and simple sugars. Phylogenetic analyses indicate that coleopteran-derived GH5_10 proteins cluster together with Chelicerata-derived ones. Interestingly, other insect-derived GH5_10 proteins cluster elsewhere, suggesting insects have several independent evolutionary origins.

## Introduction

The plant’s primary cell wall is a complex structure consisting of polysaccharides and proteins that encase and protect growing plant cells. Next to cellulose and pectins, the hemicellulose network—made of polysaccharides such as xyloglucan, xylan and mannans—is one of the main constituents of the plant’s primary cell wall [1,2]. The mannan group, which comprises pure mannan, galactomannan and glucomannan, is widely distributed among plants and algae [3] and may be part of the wall of different types of cells and tissues, such as roots, tubers, bulbs and seeds [4]. Mannans may function as seed storage and/or structural components [5,6]. For example, galactomannan, a storage polysaccharide in the endosperm cell wall of legumes, occupies up to 30% of a seed’s dry weight [7]. The backbone of the polymer is composed of mannose residues linked together by β-1,4-glycosidic bonds. In galactomannan, this backbone is substituted with α-1,6-linked galactose residues. In contrast to mannan and galactomannan, the backbone of glucomannan is made up of randomly alternating mannose and glucose residues linked together by β-1,4-glycosidic bonds [8].

Endo-β-1,4-mannanase (EC: 3.2.1.78) is a family of so-called plant-cell-wall-degrading enzymes (PCWDEs) that hydrolyze the backbone of mannan polysaccharides into oligosaccharides [9]. These enzymes are widely distributed within the tree of life: they have been found in bacteria, fungi, plants and animals [8]. According to the carbohydrate-active enzymes (CAZy) database (http://www.cazy.org/) [10], endo-β-1,4-mannanases are distributed in several glycoside hydrolase (GH) families, namely, GH5, GH9, GH26, GH44, GH113 and GH134. In metazoans, endo-β-1,4-mannanases have been identified and functionally characterized in bivalves [11,12], gastropods [13,14], Crustacea [15] and a springtail [16]. The common feature of these metazoan mannanases is that they are members of the subfamily 10 of GH5 (GH5_10), according to the current nomenclature of this gene family [17]. This subfamily of GH5 is one of the smallest described to date; as of April 2017, only 28 sequences had been found in the CAZy database. Functionally characterized enzyme members of this subfamily are all endo-β-1,4-mannanases [11,13,14,16]. Genes encoding GH5_10 have also been identified from several bacterial genomes, but to date none has been functionally characterized [18,19]. Neither fungal- nor plant-derived GH5_10 sequences are present in the CAZy database, suggesting that this subfamily of GH5 is absent from these two phyla. During a survey of transcriptomes of several herbivorous beetles member of the Phytophaga clades [20], we identified transcripts encoding GH5_10 putative mannanases in two species of the family Chrysomelidae [21]. This finding indicated that in addition to their ability to break down cellulose and pectins [22–26], some beetles of the Phytophaga clade may also possess the ability to break down mannan polysaccharides. Interestingly, an endo-β-1,4-mannanase has been characterized in a species of Phytophaga beetles—the coffee berry borer, *Hypothenemus hampei*—but it belongs to the subfamily 8 of GH5 (GH5_8) [27,28], suggesting that the ability to break down mannan polysaccharides appeared several times in the evolution of beetles of the Phytophaga clade.

Here we analyze the function and the evolutionary history of GH5_10 putative mannanases encoded by the genome of two chrysomelid beetles with different feeding habits. Larvae and adults of the green dock beetle, *Gastrophysa viridula* (Coleoptera: Chrysomelinae), feed exclusively on the foliage of dock plants (*Rumex* spp.), whereas larvae of the bean beetle, *Callosobruchus maculatus* (Coleoptera: Bruchinae), feed on the galactomannan-rich endosperm of legume seeds. Besides GH5_10 proteins, the genome of *G*. *viridula*—like other species of the subfamily Chrysomelinae—encodes GH45 and GH48 putative cellulases as well as GH28 pectinases [21,23,29]. In contrast, in *C*. *maculatus* GH5_10 proteins are only complemented by GH28 pectinases [21,23]. First, we asked whether beetle-derived GH5_10 proteins are active PCWDEs and how they have evolved. Second, we asked what their physiological function is. We demonstrate that the sole GH5_10 protein of *G*. *viridula* and one out of four GH5_10 proteins of *C*. *maculatus* are endo-β-1,4-mannanases. In addition, a second GH5_10 protein of *C*. *maculatus* has evolved to become an endo-β-1,4-xylanase, which represents the first example of such enzymatic activity in this subfamily of GH5. We show that the genes encoding GH5_10 proteins in these two distantly related chrysomelid beetles share intron positions and phases, and thus have a common origin. Finally, the phylogenetic relationships of these beetle-derived GH5_10 proteins and their counterparts found in other metazoans are quite complex, suggesting that several of the genes in this group of animals may have originated through the acquisition by horizontal gene transfer events from bacterial donors.

## Materials and methods

### Insect rearing

*Gastrophysa viridula* adults and larvae were initially collected from broad leaf dock plants (*Rumex obtusifolius*) in the vicinity of Jena, Germany (50°55'16.4"N 11°35'14.1"E). No specific permissions were required to collect *G*. *viridula*. This beetle species is not endangered or protected in any way, and the location where the beetles were collected is a park freely accessible to the public. Collected individuals were brought to the lab and larvae were raised to adulthood. Insects were reared in plastic containers on detached leaves of *R*. *obtusifolius* grown in a greenhouse. Beetles were allowed to mate and oviposit, and the offspring were used for experiments. Larvae and adults were kept under a light/dark cycle of 16:8 hours at 18°C and 13°C, respectively. *Callosobruchus maculatus* originated from a lab culture obtained from Matthew Benton (University of Cologne) and were reared in plastic containers on organic black-eyed peas at room temperature on a lab bench.

### Insect cell culture and heterologous expression

Open reading frames (ORFs) were amplified from cDNAs using gene specific primers (S1 Table) designed according to previously described GH5 sequences from *G*. *viridula* and *C*. *maculatus* [21]. The forward primer was designed to introduce a Kozak sequence at the beginning of the ORF, and the reverse primer was designed to omit the stop codon. Complementary DNAs (cDNAs) initially generated for RACE-PCR experiments as described by Pauchet and coworkers [21] were used as a template, and PCR reactions were conducted using a high-fidelity Taq polymerase (AccuPrime, Invitrogen). PCR products were cloned into the pIB/V5-His TOPO/TA (Invitrogen), in frame with the coding sequence of a V5-(His)_6_ epitope. TOP10 competent *E*. *coli* cells (Invitrogen) were transformed and plated on LB-agar dishes supplemented with 100 μg/ml ampicillin. To select for constructs correctly oriented after ligation into pIB/V5-His TOPO/TA, randomly picked colonies were checked by colony-PCR using the OpIE2 forward primer located on the vector and a gene-specific reverse primer (S1 Table). Positive clones were further cultured in 3 ml DYT-medium containing 100 μg/ml ampicillin. After plasmid isolation using GeneJET Plasmid Miniprep Kit (Thermo Scientific), the ORF of selected clones was fully sequenced in both directions using capillary sequencing to confirm that the ORF had been correctly inserted into the vector and to control that no mutation were introduced during the cloning process. Positive constructs were then transfected in *Sf*9 cells (Invitrogen) using FuGENE HD (Promega) as a transfection reagent. First, successful expression was determined by transiently transfecting three clones per construct in a 24-well plate format. After 72 h, the culture medium was harvested, and successful expression was verified by Western blot using the anti-V5-HRP antibody (Invitrogen). In order to collect enough material for downstream enzymatic activity assays, a single clone per construct was used for subsequent transfection of insect cells in a 6-well plate format. After 72 h, culture medium was harvested and centrifuged (16,000 x g, 5 min, 4°C) to remove cell debris; finally the medium was stored at 4°C until further use. Again, successful expression was verified by Western blot using the anti-V5-HRP antibody.

### Agarose diffusion assays

Enzymatic activity of the recombinant proteins was initially assessed using agarose diffusion assays. Agarose (1%) plates were prepared, containing 0.1% substrate (glucomannan, galactomannan and carboxymethyl cellulose) in 40 mM citrate/phosphate buffer pH 5.0. Galactomannan (Megazyme) was derived from Carob pods and had a Galactose to Mannose ratio of 22/78. Glucomannan and carboxymethyl cellulose were both purchased from Sigma Aldrich. Small holes were made in the agarose matrix using cut-off pipette tips, to which 10 μl of the crude culture medium of each produced enzyme was applied. After incubation overnight at 40°C, activity was revealed by incubating the agarose plate in a 0.1% Congo red solution for 2 h at room temperature followed by a washing step with 1 M NaCl for 30 min at room temperature.

### Preparation of primary cell wall from *Rumex obtusifolius* leaves

Plant cell wall was extracted from *R*. *obtusifolius* leaves according to Feiz *et al*. [30] with slight modifications. Briefly, 32 g of *R*. *obtusifolius* leaves was blended in 5 mM acetate buffer pH 4.6 and 400 mM sucrose. The plant tissue homogenate was incubated for 30 min at 4°C while being stirred and then pelleted by centrifugation for 15 min at 1000 x *g* and 4°C. The pellet was washed twice in 5 mM acetate buffer pH 4.6 containing 0.6 M and 1 M sucrose, respectively. Finally, the pellet was transferred to a 25 μm nylon net (Miracloth) and washed with 6 l of 5 mM acetate buffer pH 4.6. The resulting cell wall was ground in liquid nitrogen and then lyophilized for 48 h. To remove proteins associated with the plant cell wall, 650 mg of lyophilized cell wall material was washed twice in 25 ml of 5 mM acetate buffer pH 4.6 containing 200 mM CaCl_2_, and was then washed twice in 30 ml 5 mM acetate buffer pH 4.6 containing 1 M NaCl. For each washing step, the cell wall was homogenized by vortexing for 10 min at room temperature and subsequently centrifuged at 4000 x *g* and 4°C. Subsequently, the protein-free cell wall was washed with 3 l of double distilled water before being lyophilized. Freeze-dried plant cell wall from *R*. *obtusifolius* was rehydrated in double distilled water, resulting in a 5% stock solution. For thin layer chromatography analyses (see below), 35 μg of the 5% rehydrated PCW was incubated with 30 μl heterologously expressed GH5 from *G*. *viridula* in a 20 mM citrate/phosphate buffer pH 5.0.

### Analysis of hydrolysis reaction products by thin layer chromatography (TLC)

The culture medium of transiently transfected cells was first dialyzed against distilled water at 4°C for 24 h, using Slide-A-Lyzer Dialysis Cassettes with a 10 kDa cut-off, before being desalted with Zeba Desalt Spin Columns 7 kDa cut-off (both Thermo Scientific), according to the manufacturer’s guidelines. Samples were stored at 4°C until used. Twenty microliter enzyme assays were set up, using 14 μl of dialyzed and desalted crude enzyme extracts mixed with 4 μl of a 1% solution of substrate in a 20 mM citrate/phosphate buffer pH 5.0. The following substrates were tested: carboxymethyl cellulose, beechwood xylan (both Sigma Aldrich), glucomannan, galactomannan and xyloglucan (all Megazyme). Additionally, the manno-oligomers, D-(+) tetraose to D-(+) hexaose (Megazyme) were tested at a final concentration of 250 ng/μl. Samples were then incubated overnight at 40°C. Finally, 15 μl of the reaction was applied to TLC plates (silica gel 60, Merck) and enzymatic breakdown products were separated using the following mobile phase: butanol/glacial acetic acid/water (2:1:1). Breakdown products were revealed by spraying the TLC plates with 0.2% (w/v) orcinol in methanol/sulfuric acid (9:1) followed by heating until reaction products appeared. The reference standard contained 2 μg each of mannose, mannobiose, mannotriose, mannotetraose and mannopentaose (all Megazyme) or 2 μg each of glucose, cellobiose, cellotriose, cellotetraose and cellopentaose (all from Sigma-Aldrich) or 2 μg each of xylose (Sigma-Aldrich), xylobiose and xylotriose (both Megazyme), according to the substrate tested.

### Temperature optimum and pH optimum

To test the temperature optima, dialyzed and desalted crude enzyme extracts were incubated with 0.5% (w/v) galactomannan (GVI1), or galactomannan and carboxymethyl cellulose in parallel (CMA3), or beechwood xylan (CMA2) in 20 mM citrate phosphate buffer (pH 5.0) at different temperatures ranging from 20°C to 80°C in steps of 10°C. In detail, each enzyme assay was performed with 24 μl crude enzyme extract, 30 μl of 1% (w/v) substrate solution and 6 μl of 20 mM citrate phosphate buffer pH 5.0. Negative controls were carried out with 24 μl of distilled water instead of enzyme. The enzymatic activity was assayed at 40°C for 5 min (GVI1), 2.5 h (CMA3 against GalM), 16 h (CMA3 against CMC) and 16 h (CMA2). The amount of reducing sugars produced in these reactions was measured using the dinitrosalicylic acid (DNS) method according to Kirsch and co-workers [23]. To test for pH optima, dialyzed and desalted crude enzyme extracts were incubated with their respective substrate as described above and assayed in 20 mM citrate phosphate buffers ranging from pH 2.0 to 9.0 as well as in 20 mM sodium carbonate buffer pH 10.0. The amount of reducing sugars produced in these reactions was measured using the dinitrosalicylic acid (DNS) method as described above. Each reaction was carried out in triplicate.

### Preparation of double-stranded RNA and off-target prediction

Primers for RNA interference (RNAi) experiments were designed for *G*. *viridula* GH5 (GVI1) and GFP used as controls, yielding a 300 bp fragment and a 379 bp fragment, respectively (S1 Table). To predict potential off-target effects, the sense and anti-sense RNA strands were diced *in silico* into all possible 21 bp fragments using an in-house algorithm. The resulting siRNAs were searched against our *G*. *viridula* larval gut transcriptome [21], using previously described parameters [31]. A siRNA was considered off-target if the resulting hit was equal to or higher than 21 bp by allowing one mismatch. Gene fragments were amplified from sequenced recombinant plasmids containing GVI1 or GFP. The amplicons were gel-purified using Zymoclean Gel DNA recovery Kit (Zymo Research). To obtain double-stranded RNA (dsRNA), the purified PCR product was used as a template for *in vitro* transcription using the MEGAscript RNAi kit (Ambion), following the manufacturer’s instructions. To remove residual DNA contamination, the resulting dsRNA was nuclease-digested using TURBO^™^ DNase (Thermo Scientific), and then purified and recovered in 150 μl injection buffer (3.5 mM Tris–HCl, 1 mM NaCl, 50 nM Na2HPO4, 20 nM KH2PO4, 3 mM KCl, 0.3 mM EDTA, pH 7.0). The quantity of dsRNA was estimated using a spectrophotometer (NanoDrop ND-1000, Peqlab Biotechnology), and its quality was assessed by gel electrophoresis.

### Injection of dsRNA and assessment of RNAi efficiency

Early second-instar *G*. *viridula* larvae were injected dorsally with 50 nl (150 ng) of target dsRNA into the metathorax, using a Nanoliter 2010 Injector (World Precision Instruments) attached to a three-dimensional micromanipulator, and were then put onto fresh *R*. *obtusifolius* leaves. To record weight gain and mortality, five animals per replicate were injected with a total of six replicates for each target gene. To analyze gene expression and enzymatic activity, three animals per replicate were injected with a total of six replicates for each target gene. In addition to larvae injected with dsRNA targeting GFP, a non-injected control was also included. For quantitative PCR and enzymatic activity analyses, larvae were collected at days 1, 4 and 8 post injection. Whole larvae were crushed in liquid nitrogen and separated in half. One aliquot was used for total RNA preparation, the other for protein extraction.

Total RNA was isolated using innuPREP RNA Mini Kit (Analytik Jena), following the manufacturer’s protocol. The resulting RNA was then subjected to DNase digestion (Ambion), and its quality was subsequently checked using the RNA 6000 Nano LabChip kit on a 2100 Bioanalyser (both Agilent Technologies). Total RNA was used as a template to synthesize cDNAs using the Verso cDNA synthesis kit (Thermo Scientific). The resulting cDNA samples were then used for real-time qPCR experiments, which were performed in 96-well hard-shell PCR plates on the CFX Connect Real-Time System (both Biorad). All reactions were carried out using the 2-Step QPCR SYBR Kit (Thermo Scientific), following the manufacturer’s instructions. Primers were designed using Primer3 (version 0.4.0) (S1 Table). The specific amplification of each transcript was verified by dissociation curve analysis. A standard curve for each primer pair was determined in the CFX Manager (version 3.1) based on Cq-values (quantitation cycle) of qPCRs run with a dilution series of cDNA pools. The efficiency and amplification factors of each qPCR, based on the slope of the standard curve, were calculated using an integrated efficiency calculator of the CFX manager software (version 3.1). The sequence of the transcript encoding ribosomal protein S3 (*RPS3*), extracted from our *G*. *viridula* larval gut transcriptome [21], was used as a reference for all qPCR experiments, and the abundance of GVI1 transcripts was expressed as RNA molecules per 1000 RNA molecules of *RPS3*.

To directly compare GH5 transcript abundance to GH5 enzymatic activity in RNAi-treated *G*. *viridula*, crushed and frozen material was suspended in 40 mM citrate/phosphate buffer at pH 5.0 containing a protease inhibitor cocktail (Complete EDTA-free, Roche). Then, the samples were centrifuged (10 min, 16000xg, 4°C), and the supernatant was collected and stored at 4°C until further use. Protein concentration was estimated by Bradford Protein Assay (Bio-Rad). Enzymatic activity assays were carried out using the DNS method as described above, using 2 μg of extracted proteins in the reaction. Alternatively, 0.5 μg total extracted proteins were prepared for zymogram analysis by diluting the sample in Laemmli buffer without any reducing agent. Samples were run on a 12.5% SDS-PAGE gel containing 0.1% (w/v) galactomannan. Electrophoresis was carried out at 4°C using pre-chilled running buffer. Gels were then washed three times in a 2.5% Triton X-100 solution for 15 min, each at 4°C, before being equilibrated in the reaction buffer (50 mM citrate/phosphate buffer pH 5.0) for 16 h at 4°C, followed by a 1 h incubation at 40°C. The gels were then incubated in a 0.1% (w/v) Congo red solution before being destained in 1 M NaCl until pale activity zones appeared against a dark red background.

Two life history traits were recorded after larvae were injected with dsRNA. First, larvae (we used groups of five insects per replicate, six replicates in total) were weighed on day 1and day 8 post injection. Then, growth rate was calculated using the formula “*Growth rate = Log*_*10*_*(Final weight)-Log*_*10*_*(Initial weight)/ Time (days)*”. Finally, mortality was recorded at the end of the experiment.

### Tissue-specific gene expression

Late-instar *G*. *viridula* larvae, actively feeding on leaves of *R*. *obtusifolius*, as well as late-instar *C*. *maculatus* larvae, actively feeding inside black-eyed peas, were used for RNA extraction. Larvae were cut open from abdomen to head, and the complete gut was removed and stored separately from the rest of the body. Dissection and storage were carried out in RL solution (Analytik Jena). Three biological replicates were sampled, each containing three larvae. RNA extraction, generation of cDNAs and subsequent real-time qPCR experiments were performed as described above. Primers used for these experiments are listed in S1 Table.

### Statistical analyses

If not otherwise stated data were analyzed in R version 3.2.0 [32]. Statistical analyses of gene expression over time were performed as follows: The influence of GVI1 RNAi treatment (iGH5) over time RNAi treatment and time used as categorical explanatory variables on GVI1 transcript abundance was investigated using the generalized least squares method (gls from the nlme library [33]) to account for the variance heterogeneity among the residuals. The varIdent variance structure was used, with a different variance for the combination of treatment and time (varIdent (form = ~1|combination of [treatment and time])). The influence of the explanatory variables was determined by sequentially removing explanatory variables starting with the full model and comparing the simpler model to the more complex one, using a likelihood ratio test [34]. Differences between factor levels were determined by factor level reduction [35]. The influence of RNAi treatment on the enzyme activity over time was analysed with a two-way ANOVA. The Tukey HSD test was performed in order to find differences between the groups. To compare weight gain over time in RNAi-treated larvae, we calculated the relative growth rate for the period of 8 days and analyzed the data in SigmaPlot version 11.0 (Systat Software) using a one-way ANOVA. Differences in mortality were analyzed using the equality of proportions–test. Differences in tissue-specific gene expression were analyzed with paired t-tests again in SigmaPlot Version 11.0.

### Gene structure determination

The GVI1 ORF was amplified by PCR from genomic DNA using gene-specific primers (S1 Table). Genomic DNA was prepared from a single *G*. *viridula* male beetle using the QIAamp DNA micro kit (Quiagen), following the manufacturer’s instructions. The PCR product was then cloned into the pCR4 TOPO TA vector (Invitrogen), followed by the transformation of TOP10 competent *E*. *coli* cells (Invitrogen). Cells were then plated on LB-agar dishes supplemented with 100 μg/ml ampicillin. To select for constructs harboring the sequence of interest, randomly picked colonies were checked by PCR using M13 forward and reverse primers. Plasmid DNA was prepared from positive clones using the GeneJET Plasmid Miniprep Kit (Thermo Scientific). The sequence of the GVI1 ORF was deduced from three independent clones after capillary sequencing. Sequences corresponding to the genes encoding CMA1, CMA2, CMA3 and CMA4 were retrieved from a draft genome assembly of *C*. *maculatus* made publicly available (http://www.beanbeetles.org/genome/). The intron/exon structure was determined for each gene using Splign [36].

### Amino acid alignment and phylogenetic analyses

All sequences corresponding to GH5_10 proteins present in the carbohydrate-active enzymes (CAZy) database [37] as of February 1, 2017, were retrieved. Due to the paucity of GH5_10 sequences available, extra searches were conducted in bacterial and fungal genomes available through the genome portal of the Joint Genome Institute (http://jgi.doe.gov/) as well as in transcriptome shotgun assemblies available at Genbank (https://www.ncbi.nlm.nih.gov/genbank/tsa/). A description of the sequences can be found in S2 Table. All obtained sequences were analyzed for the presence of a signal peptide and extra protein domains other than the GH5 domain using InterProScan version 61.0. Once such features were identified, they were removed from the collected protein sequences, and only the GH5 domain was conserved for amino acid alignment. Amino acid alignments were carried out using MUSCLE version 3.7 on the Phylogeny.fr web platform (http://www.phylogeny.fr) [38], and were inspected and corrected manually when needed. Maximum-likelihood-inferred phylogenetic analyses were conducted in MEGA6 [39]. The best model of protein evolution was determined in MEGA6 using the ‘find best DNA/protein models’ tool. The best model was the ‘Le and Gascuel’ (LG) model, incorporating a discrete gamma distribution (shape parameter = 5) to model differences in evolutionary rates among sites (+G) and a proportion of invariable sites (+I). The robustness of each analysis was tested using 1,000 bootstrap replicates.

## Results

### Characterization of *G*. *viridula* and *C*. *maculatus* GH5_10 proteins reveals distinct enzymatic activities

**Fig 1:**
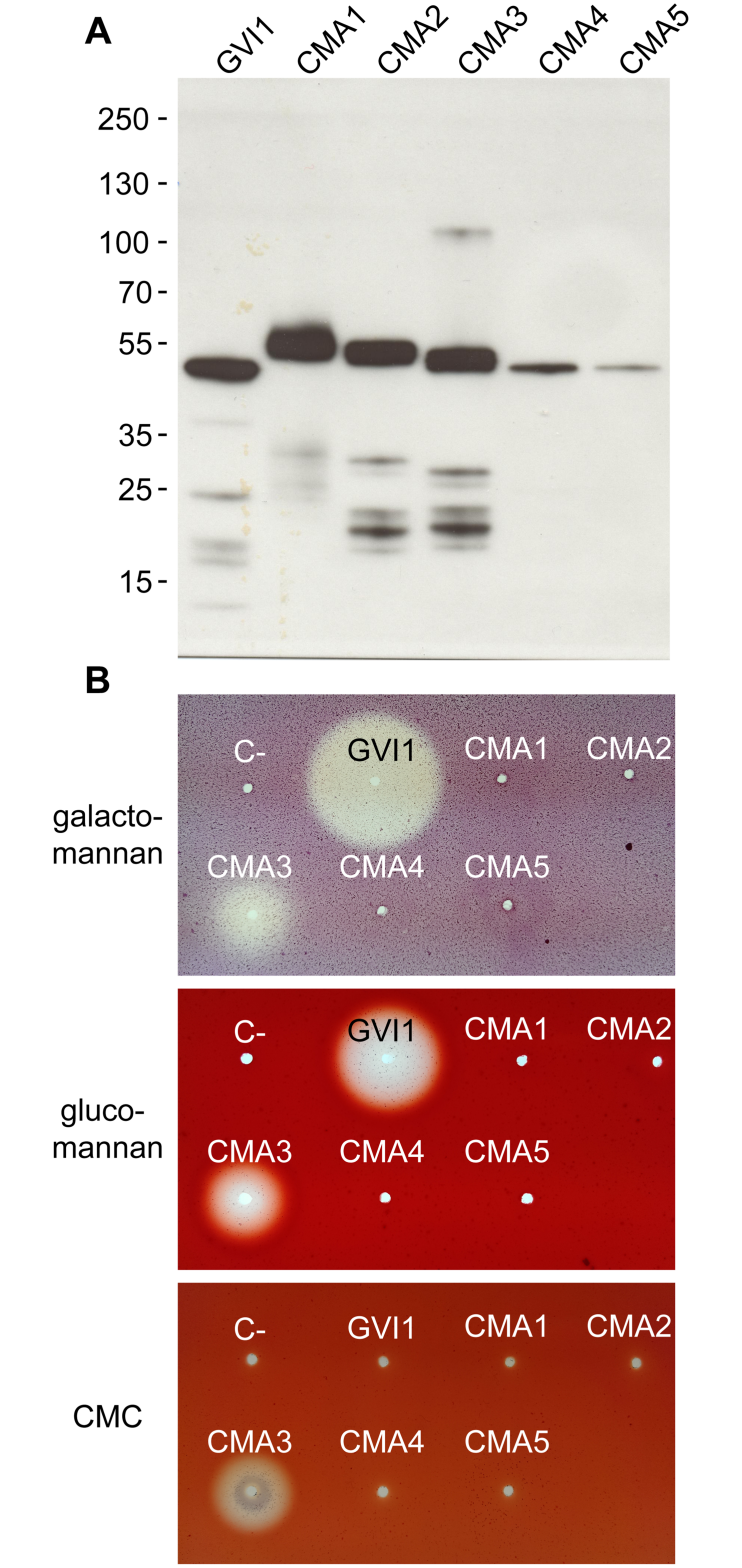
Heterologous expression of GH5_10 proteins from *G*. *viridula* and *C*. *maculatus* in *Sf*9 insect cells. (A) GH5_10 cDNAs cloned into an expression vector in frame with a V5/(His)_6_ epitope were transfected into *Sf*9 cells. The culture medium of transfected cells was collected 72 hours post transfection and samples were subjected to Western blot. An anti-V5-HRP antibody was used for detection and the blot was revealed using chemiluminescence. Molecular weight markers are indicated next to the Western blot. (B) The culture medium of transfected cells was applied to agarose plates containing 0.1% substrate in McIllvain buffer pH 5.0, and plates were incubated for 16 hours at 40°C. Activity halos were revealed after staining with Congo red.

Full-length amplicons of target GH5_10 transcripts were cloned into a pIB-V5/His TOPO vector and transiently expressed in insect *Sf*9 cells. Validation that GH5_10 proteins were successfully expressed and secreted into the culture medium was made by Western blot; heterologous proteins had an apparent molecular weight of 45 to 55 kDa close to their theoretical expected size ranging from 41.7 to 43.2 kDa (Fig 1A). To test whether these GH5_10 proteins were enzymatically active, we initially analyzed crude enzyme extracts on agarose diffusion plates containing various plant cell wall polysaccharides as substrates (Fig 1B). *Gastrophysa viridula* GH5-1 (GVI1) and *C*. *maculatus* GH5-3 (CMA3) exhibited activity halos on plates containing galactomannan (GalM) and glucomannan (GluM). In addition, CMA3 showed activity halos on plates containing carboxymethyl cellulose (CMC).

**Fig 2:**
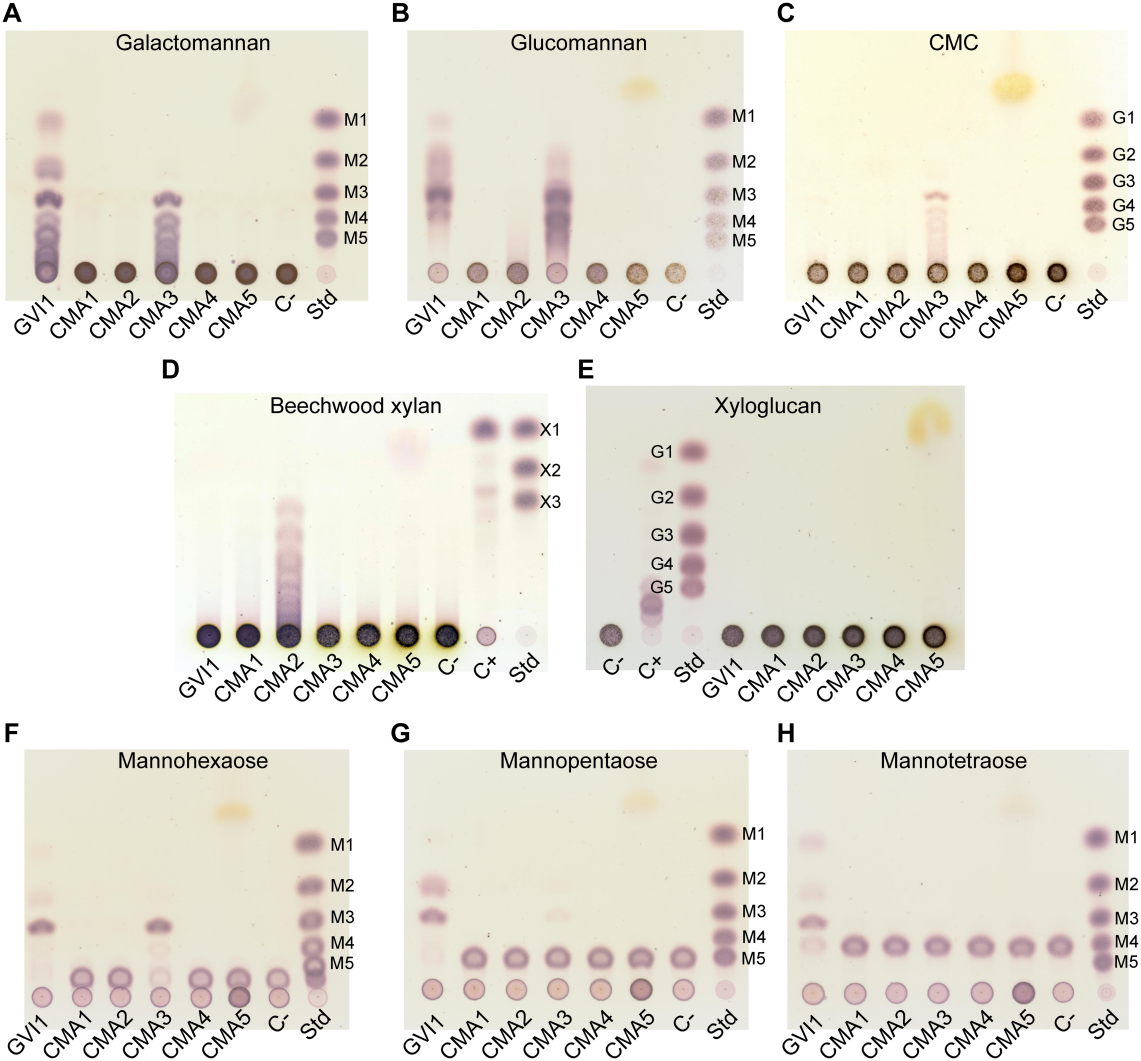
Thin-layer chromatograms of beetle GH5_10 assays against a range of plant cell wall polysaccharides and mannan oligomers. (A) Heterologously expressed *G*. *viridula* and *C*. *maculatus* GH5_10 proteins were incubated with galactomannan. GVI1 releases mannose, mannobiose, mannotriose and larger oligomers. CMA3 releases mannotriose and larger oligomers. (B) The same proteins incubated with glucomannan. GVI1 and CMA3 release a range of oligomers, which proved difficult to resolve on TLC. (C) The same proteins incubated with carboxymethylcellulose (CMC). CMA3 releases cellotriose and larger oligomers. (D) The same proteins incubated with beechwood xylan. CMA2 releases xylotriose and larger oligomers. (E) The same proteins incubated with xyloglucan. None of the proteins showed activity against this substrate. (F) The same proteins incubated with mannohexaose. Both GVI1 and CMA3 release mannotriose. (G) The same proteins incubated with mannopentaose. GVI1 releases mannotriose and mannobiose. (H) The same proteins incubated with mannotetraose. GVI1 releases mannose, mannobiose and mannotriose. Standards (Std) used are mannose to mannopentaose (M1 to M5), glucose to cellopentaose (G1 to G5), xylose to xylotriose (X1 to X3). A negative control was introduced (C-) to which no enzyme was added. A positive control (C+), which is composed of a commercial cellulase preparation from *Trichoderma reesei* incubated with the corresponding substrates, was included in the TLCs of xylan and xyloglucan activity assays.

To further analyze the enzymatic properties of beetle-derived GH5_10 proteins, we used in-tube assays with an array of cellulosic and hemicellulosic poly- and oligosaccharides, and analyzed them by TLC (Fig 2). GVI1 exhibited activity against GalM and GluM (Fig 2A and 2B). GalM breakdown products consisted mainly of trimers and larger oligomers and, to a lesser extent, monomers and dimers of mannose. GluM breakdown products seemed to be trimers and tetramers and, to a lesser extent, dimers and monomers (Fig 2A and 2B). Compared to GalM breakdown products, however, these oligomers appear to be far less resolved on TLC. Most likely, this discrepancy in the resolution of breakdown products lies in the chemical nature of both substrates. GalM is a polysaccharide consisting of a pure mannose backbone decorated with evenly distributed galactose moieties, whereas GluM is a straight-chain polysaccharide consisting of a backbone that alternates unevenly between mannose and glucose moieties with occasional branching. Thus, we believe that the heterogeneous structure of GluM leads to inconsistently sized breakdown products which appear as smears on TLC. We then tested the ability of GVI1 to cleave several mannan oligomers (Fig 2). GVI1 cleaved mannohexaose into mannotriose (Fig 2F), and mannopentaose into mannotriose and mannobiose (Fig 2G). The smallest mannan oligomer that GVI1 could cleave was mannotetraose, resulting in the breakdown products mannotriose, mannobiose and mannose (Fig 2H).

Like GVI1, CMA3 exhibited activity against galactomannan and glucomannan (Fig 2A and 2B), but CMA3 was also able to break down CMC (Fig 2C). The main breakdown products accumulating after GalM and CMC degradation are the corresponding trioses and the larger oligomers. As we observed for GVI1, GluM breakdown products did not resolve very well on TLC. The smallest oligomer that CMA3 was able to cleave was mannohexaose, releasing mannotriose (Fig 2F). The results obtained for GVI1 and CMA3 on TLC confirmed the activity observed in agarose plate assays. Our data strongly indicate that GVI1 and CMA3 are endo-β-1,4-mannanases, with CMA3 also acting as endo-β-1,4-glucanase.

Unexpectedly, TLC experiments allowed us to detect a second enzymatically active GH5_10 protein in *C*. *maculatus* (CMA2), which was able to degrade beechwood xylan (Fig 2D). The breakdown products generated by CMA2 were xylotriose and larger oligomers of xylan, which suggests that this enzyme acted as an endo-β-1,4-xylanase. GH5 family members harboring xylanase activity have been described in bacteria [40,41] and, recently, in cerambycid beetles [25,26]. However, these xylan-degrading GH5 enzymes were encoded by distinct GH5 subfamilies (GH5_2, _4 and _21) [17]. CMA2 represents the first example of a GH5_10 protein harboring endo-β-1,4-xylanase activity.

None of the tested heterologously expressed proteins showed activity against xyloglucan (Fig 2E). No activity either on plates or on TLC was observed for *C*. *maculatus* GH5-1, -4, -5 (CMA1, CMA4, CMA5) on any of the substrates tested. An alignment of the amino acid sequences including all coleopteran-derived GH5_10 proteins together with two additional sequences of proteins with known crystal structures [11,42] showed that the two catalytic glutamate residues are conserved, and confirmed that no dramatic substitutions of active site residues occurred between the two reference sequences and the sequences derived from beetles (S1 Fig). These patterns may indicate that CMA1, CMA4 and CMA5 are active enzymes but that their substrate has not yet been discovered or, alternatively, that they lost their activity due to mutations in other functionally important sites.

### Investigation of optimal pH values and temperatures for coleopteran GH5_10 enzymes

**Fig 3:**
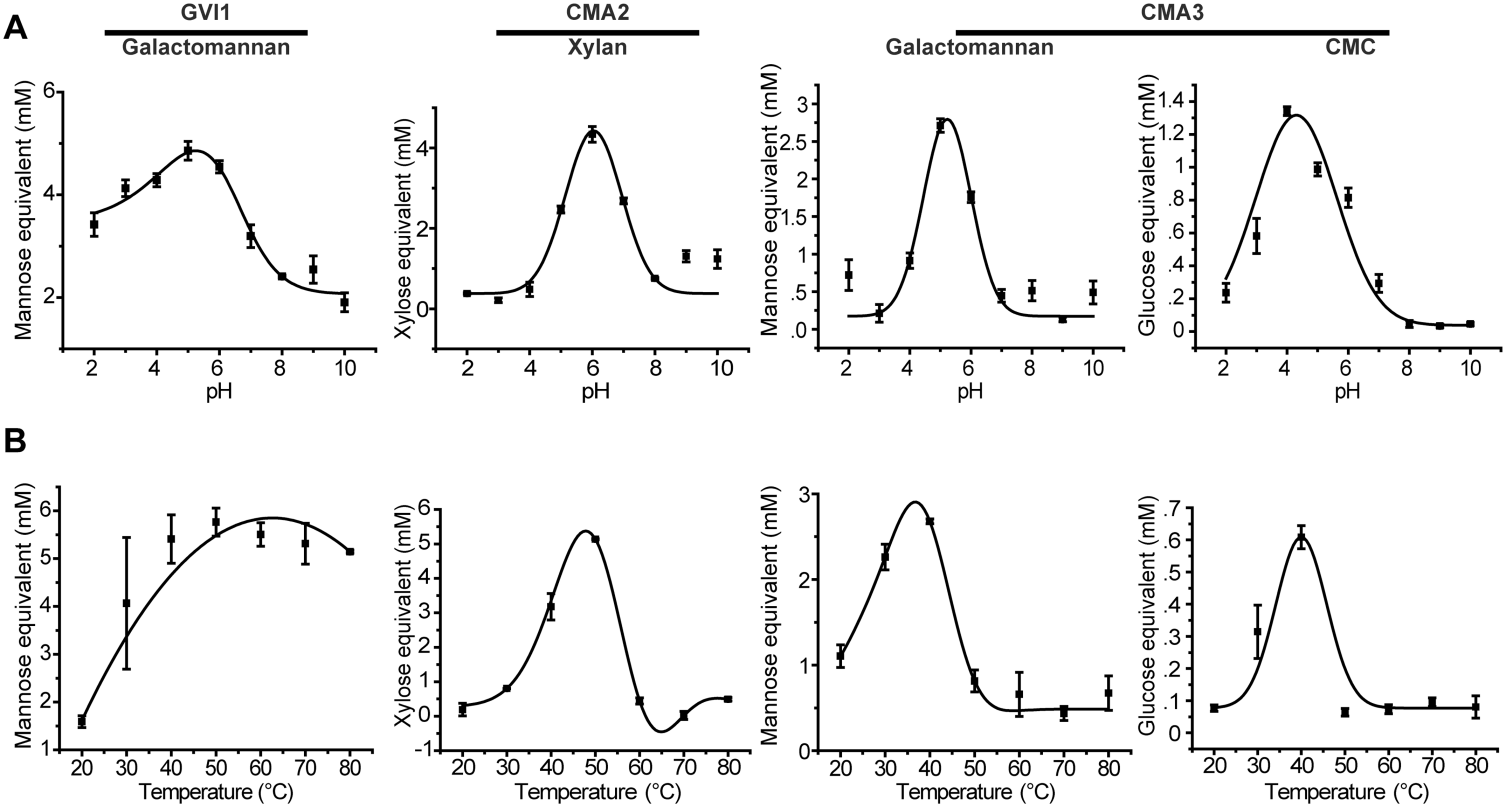
Determining the optimal pH values and temperatures of the enzymatically active GH5_10 proteins. (A) GVI1, CMA2 and CMA3 were incubated with their respective substrates at various pH values, ranging from 2.0 to 10.0. (B) The same proteins were incubated with their respective substrates at various temperatures, ranging from 20 to 80°C. The amount of reducing sugars released was determined by DNS assay and converted into millimolar (mM) of sugar monomer equivalent. The results are the means of three independent replicates ±SEM. The substrates used were galactomannan for GVI1 and CMA3, beechwood xylan for CMA2 and carboxymethylcellulose (CMC) for CMA3.

The enzymatic performance of GVI1 was monitored using galactomannan as a substrate. GVI1 performed best at acidic pH values with an optimum around pH 5.0 (Fig 3A) and a temperature optimum close to 50°C (Fig 3B). As CMA3 was the only enzyme we discovered exhibiting activity against three substrates—namely, GalM, GluM and CMC—we chose to test CMA3 against GalM and CMC in parallel. The optimal pH value for CMA3 using GalM as a substrate was 5.0, and the corresponding optimal temperature was around 40°C (Fig 3A and 3B). The optimal pH value for CMA3 tested against CMC was close to 4.0 and the corresponding optimal temperature, around 40°C (Fig 3A and 3B). The enzymatic performance of CMA2 was monitored using beechwood xylan as a substrate. The pH optimum for CMA2 was determined to be around 6.0 and the optimal temperature was approximately 50°C. In summary, each enzyme analyzed performed best under acidic conditions, which correlates well to the pH conditions of the gut lumen in related beetle species [43].

### Tissue-specific gene expression and gene silencing of the *G*. *viridula* GH5_10 gene

To learn where the genes encoding GH5_10 family members are expressed in *G*. *viridula* and *C*. *maculatus*, we performed quantitative RT-PCR on midgut tissue and on the rest of the body. Transcripts encoding all GH5_10 proteins in both species are significantly more abundant (statistical values see S3 Table) in the midgut tissue, whereas almost no transcripts were detected in the rest of the body, reinforcing the fact that these GH5_10 proteins have a digestive function (S2 Fig).

To investigate how important GH5_10 are for the biology of these beetles, we used dsRNA-mediated silencing of the expression of the gene encoding GVI1 in *G*. *viridula* and analyzed the subsequent genotype and phenotype. We decided to perform RNAi experiments only with G. viridula. In fact RNAi is not really possible to perform with C. maculatus mainly due to the biology of this beetle species. As mentioned earlier, C. maculatus larvae develop inside legume seeds. One would have to remove the larvae out of the seeds to inject the dsRNA and then put them back into a legume seed which is practically not possible.

**Fig 4:**
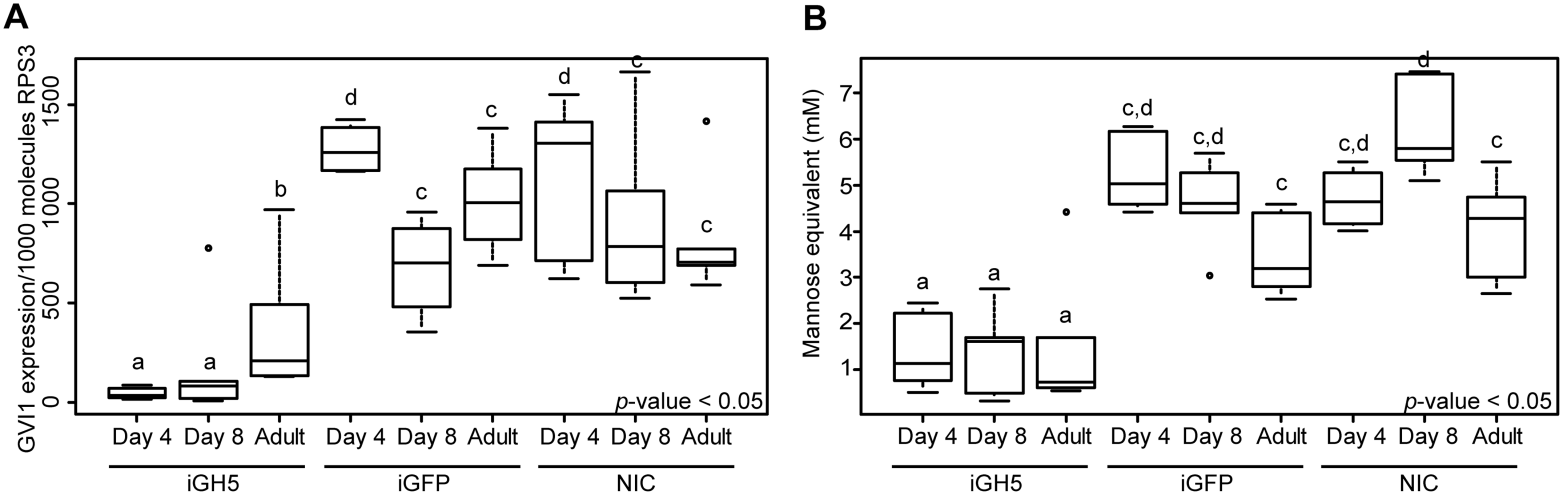
Knockdown of the expression of the gene encoding GVI1 by RNA interference. Early second-instar larvae of *G*. *viridula* were injected with double-stranded RNA (dsRNA), targeting GVI1 (iGH5) or targeting GFP (iGFP) as a control. A non-injected control (NIC) was also included. Larvae were collected on days 4 and 8 post injection. Newly emerged adults were also collected. Groups of three insects (six replicates per treatment) were snap-frozen in liquid nitrogen before being ground into a fine powder. Half of the powder was used for total RNA preparation and subsequent quantitative RT-PCRs, and the other half was used in enzyme assays. (A) The expression of the gene encoding GVI1 was assessed in the different treatments by quantitative RT-PCR. The gene expression is given as copy number per 1000 RNA molecules of RPS3. (B) Quantification of the mannanase activity in the same treatments. The amount of reducing sugars released was determined by DNS assay and converted into millimolar (mM) of mannose. The different letters on top of each box plot indicate significant differences (P < 0.05). For details on the statistics, please refer to the Materials and Methods section.

First, we examined gene expression levels of GVI1 at several time points after the injection of dsRNA (Fig 4A). We managed to significantly knock down the expression of the GVI1 gene (iGH5) compared to insects injected with dsRNA targeting GFP as control (iGFP) (likelihood ratio = 59.634, p < 0.001). The changes of the expression levels over time differed between treatments (likelihood ratio = 21.114, p<0.001). More precisely, we were able to reduce the expression of the GVI1gene up to 96.6% at day 4 post injection, 70.8% at day 8 post injection and 64.7% in adults compared to iGFP control animals. In general, our knockdown of the expression of the GVI1 gene using dsRNA proved to be stable and lasted through the adult stage (Fig 4A). Second, we measured the mannanase activity of guts dissected from iGH5 larvae at several time points. We found that levels of mannanase activity in iGH5 insects were significantly reduced compared to iGFP and NIC control insects (Fig 4B) (F = 71.361, p< 0.001). The changes of enzymatic activity over time differed between treatments (F = 3.499, p = 0.014) and was reduced to 73.96% at day 4 post injection, 70.32% at day 8 post injection and 57.93% in adults compared to iGFP control insects. Again, the reduction of mannanase activity remained stable in the gut of iGH5 insects from the larval to the adult stage (Fig 4B).

Although the reduction of mannanase activity in iGH5 insects was substantial and correlated with the knockdown of the expression of the gene encoding GVI1, it did not reach the same level (that is, ca. 95% reduction). Therefore we asked whether GVI1 is the only mannanase present in the gut of *G*. *viridula*. To answer this question, we performed zymogram analyses using galactomannan as a substrate, and compared iGH5 to iGFP and non-injected control insects (S3 Fig). A single band harboring mannanase activity could be seen in all samples. The intensity of the band in iGH5 protein samples was strongly reduced compared to the intensity of the band from the control insects, indicating (i) that GVI1 is the sole endo-β-1,4-mannanase present in the gut fluid of *G*. *viridula*; and (ii) that there is still a non-negligible amount of this enzyme even after RNAi.

**Fig 5:**
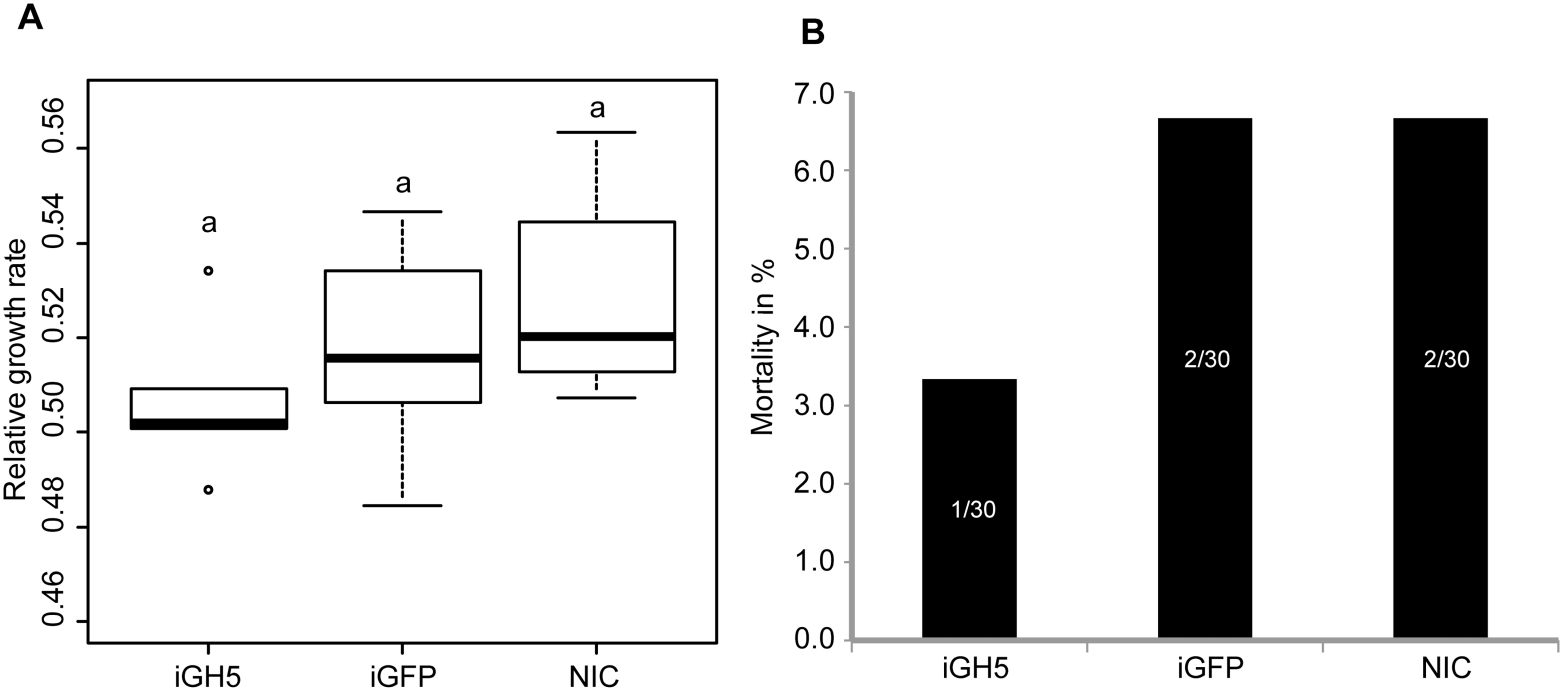
Effects of knocking down the expression of the gene encoding GVI1 on growth rate and mortality. Early second-instar larvae of *G*. *viridula* were injected with double-stranded RNA (dsRNA) targeting GVI1 (iGH5) or targeting GFP (iGFP) as a control. A non-injected control (NIC) was also included. Larvae were collected at days 4 and 8 post injection. Newly emerged adults were also collected. (A) Groups of five insects (six replicates per treatment) were weighed on day 1 and day 8 after they were injected with dsRNA, and growth rates were calculated. A one-way ANOVA statistical test was applied to the data. (B) The number of dead larvae per treatment was recorded during the eight days of the experiment. Mortality data were analyzed using the equality of proportions–test.

We then monitored two life history traits (growth rate and mortality) and compared them between iGH5 insects and both iGFP and non-injected control insects over several days post injection (Fig 5). We chose day 8 post injection for our analysis as this was the last day larvae actively fed. Although our data suggested iGH5 insects tend to grow more slowly compared to control insects, no statistically significant differences could be documented (Fig 5A) (F = 1.875, p = 0.188). Additionally, we saw no significant differences in the mortality of larvae injected with GH5 dsRNA compared to control larvae (Fig 5B, χ^2^ = 0.423, p = 0.809). In summary, although we managed to knock down the expression of the gene encoding GVI1 and reduce levels of mannanase activity, we observed no differences in growth and mortality. As a result, we believe that GVI1 may not have a primary digestive function but is more likely an accessory enzyme.

### Significance for *G*. *viridula* to express a mannanase

Taking into account that the amount of mannan polysaccharides in the primary cell wall of plant leaves is usually comprised between 2 and 5% [44], we wondered whether, the action of GVI1 could release mannan oligomers from the cell wall of *Rumex* leaves. To test this hypothesis, we isolated primary cell wall from leaves of *R*. *obtusifolius* free of proteins and incubated it with GVI1 heterologously expressed in *Sf*9 cells; then we visualized the results of this experiment on TLC (S4 Fig). We observed the presence breakdown products in cell wall samples treated with GVI1, whereas no oligomers could be detected in cell wall samples incubated alone, indicating that the primary cell wall of *R*. *obtusifolius* leaves possesses polysaccharides that can be broken down by GVI1 in the gut of *G*. *viridula* beetles.

### Phylogenetic analysis and evolution of GH5_10 proteins

**Fig 6:**
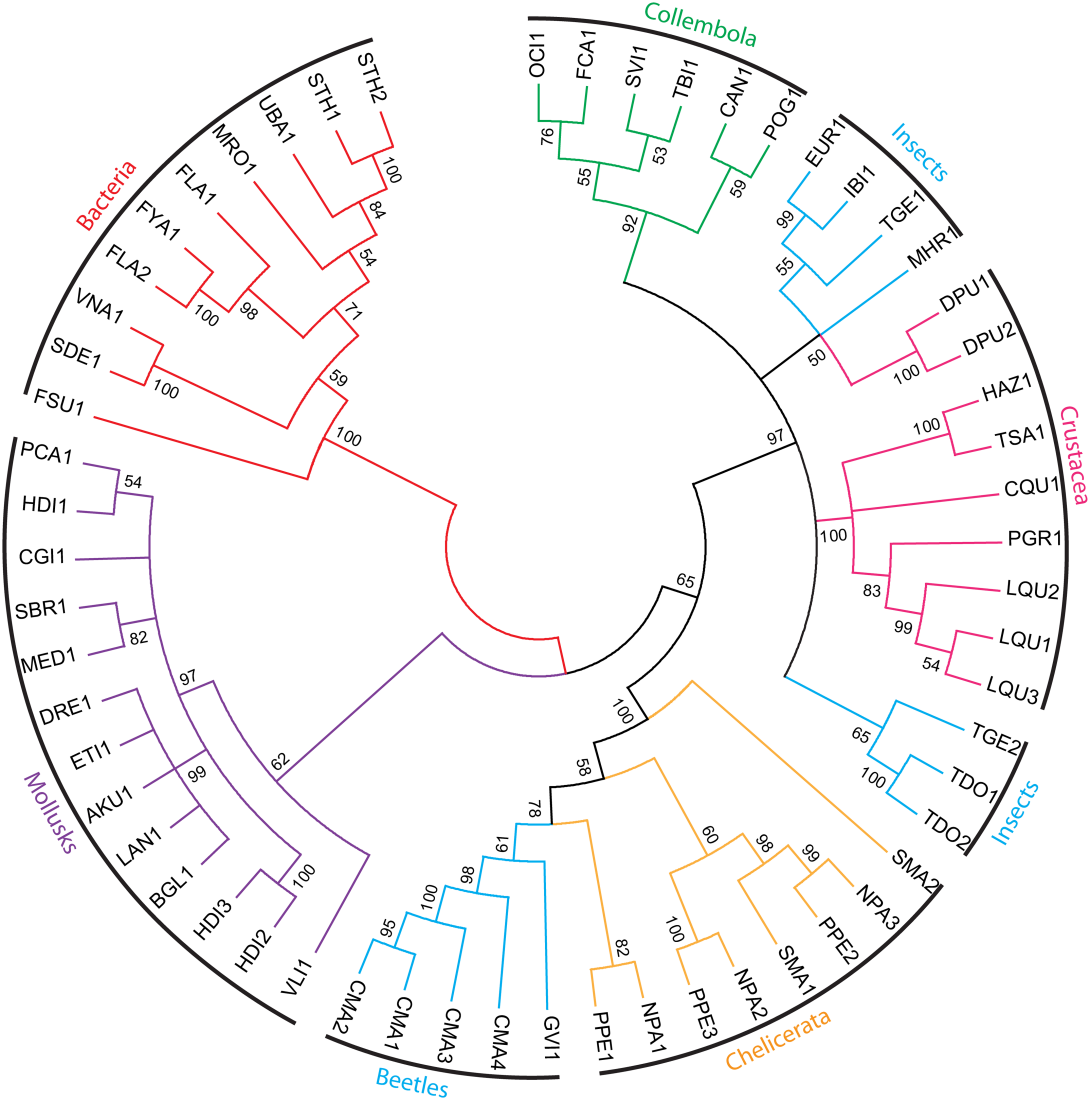
Phylogenetic relationships among beetle GH5_10 proteins and other animals and bacteria. A maximum-likelihood-inferred phylogeny is shown which compares the predicted amino acid sequences of the GH5_10 proteins from *G*. *viridula* and *C*. *maculatus* described here with their other animal and bacterial counterparts. Bootstrap support values (1000 replicates) are indicated at corresponding nodes. When the bootstrap support value of a given node was below 50, the corresponding node was condensed. Details of the sequences used for the analyses as well as accession numbers are provided in the electronic supplementary material, S2 Table. Branches in blue correspond to insect proteins; branches in red to bacterial proteins; branches in purple to mollusk proteins; branches in orange to chelicerate proteins; branches in green to collembolan proteins; and branches in pink to crustacean proteins.

To clarify the evolutionary history of GVI1 and CMA1–4 (CMA5 was excluded from the analysis as we believe it is an allele of CMA4), we reconstructed their molecular evolution in a phylogenetic analysis. We performed extensive searches of GH5_10 genes in various databases, including the CAZy database (http://www.cazy.org; [37]), as well as in publicly available genome and transcriptome assemblies. We identified GH5_10 genes in several other insects (Zygentoma, Archaeognatha, Ephemeroptera) and also in Collembola, Crustacea, Chelicerata of the family Oribatidae, mollusks and bacteria (See S2 Table). According to current data, GH5_10 genes seem to be absent in plants and fungi. Amino acid sequences collected from our search were first aligned with each other and a maximum-likelihood-inferred phylogenetic analysis was performed, showing that the coleopteran-derived GH5_10 proteins clustered in a highly supported clade together with Oribatidae (Chelicerate)-derived proteins (Fig 6). Interestingly, other insect-derived GH5_10 proteins do not cluster together with the coleopteran proteins but form a well-supported clade together with crustacean- and collembolan-derived GH5_10 proteins. This heterogeneous distribution of insect GH5_10 proteins may hint at several independent evolutionary origins for this gene family in insects, indicating the likelihood of several potential horizontal gene transfer events from bacterial donors.

We investigated beetle-derived GH5_10 encoding genes in more detail by examining intron-exon structures (S5 Fig). We investigated three full-length (GVI1, CMA3, CMA4) and two partial genomic sequences (CMA1, CMA2). For that, we PCR-amplified the gene encoding GVI1 from genomic DNA, and we acquired the structure of the CMA GH5_10 genes from a draft genome sequence. Two introns were identified in the gene encoding GVI1. The position of these two introns is conserved in the genes encoding GVI1 and the CMA GH5_10 genes. We also found extra introns in the CMA sequences. Not only do the gene encoding GVI1 and the CMAs GH5_10 genes share two intron positions, but the phase of these introns is also conserved, suggesting that the most recent common ancestor of *G*. *viridula* and *C*. *maculatus* may have possessed one or several GH5_10 genes in its genome. Also, the presence of introns in all target genes confirmed that these genes are endogenous to the beetles’ genomes.

## Discussion

We previously reported that the transcriptomes of two phytophagous beetles, *G*. *viridula* and *C*. *maculatus*, harbor transcripts encoding GH5_10 putative mannanases [21]. Here, we demonstrated that at least some of these GH5_10 proteins are indeed enzymatically active on plant cell wall polysaccharides, implying that they have a digestive function. According to our data, the gene encoding GVI1 is expressed in the gut and the corresponding protein is secreted into the gut lumen. The same is also true for the GH5_10 genes and corresponding proteins found in *C*. *maculatus*. A transcriptome analysis of *C*. *maculatus* indicated that the transcripts corresponding to CMA3 and CMA4 were expressed specifically in larval gut tissue [45]. In addition, the corresponding proteins, as well as CMA2, were identified in a proteome analysis of *C*. *maculatus*, indicating that they had been secreted into the gut lumen [46]. Clearly, these enzymes fulfill a digestive function in these beetles.

According to our data, GVI1 and CMA3 are mannanases, which mainly break down galactomannan, and most likely also mannan. The ability of these enzymes to break down glucomannan as well is most likely due to their ability to cleave β-1,4-bonds of two adjoining mannose residues in the glucomannan backbone. The additional activity observed for CMA3 on carboxymethylcellulose may be explained by the ability of this enzyme to partially cleave β-1, 4-bonds between two glucose residues. This ability is most likely due to the fact that glucose is an isomer of mannose, and the high molecular similarity of both polysaccharides might lead to substrate recognition and subsequent cleavage by CMA3. However, we would like to point out that no such secondary enzymatic activity on carboxymethylcellulose was observed for GVI1. Thus, the ability of a GH5_10 mannanase to act as cellulase is the exception rather than the rule for this family of enzymes. In addition, we know that *G*. *viridula* encodes members of the GH45 family which are absent in *C*. *maculatus* [21]. Most GH45 proteins characterized from phytophagous beetles are endo-β-1,4-glucanases that can break down amorphous cellulose [22,25,26,47–49]. Thus, CMA3 may have evolved to break down amorphous cellulose on top of mannans to adapt to the loss of genes encoding GH45 cellulases present in other subfamilies of Chrysomelidae. Notably, bi-functional mannanase-cellulase enzymes such as CMA3 are rare among GH5 enzymes and were recorded only once for a GH5_1 protein in *Ruminococcus albus* (which is only able to cleave CMC and GluM as secondary activity to lichenan degradation) [50]. To our surprise, we discovered that CMA2 has lost its ability to degrade mannans and has evolved to break down xylan, another hemicellulosic polysaccharide. Xylanase activity in coleopteran species mediated by GH5s has so far been shown only for two cerambycid beetles, but those cerambycid-derived enzymes belonged to a different GH5 subfamily than those investigated here [25,26]. Additionally, xylanases have been identified in the coffee berry borer, *Hypothenemus hampei* [51], and the mustard leaf beetle, *Phaedon cochleariae* [52], but those enzymes belonged to entirely different GH families (GH10 and GH11, respectively).

As cellulolytic and pectolytic enzymes are widely distributed among phytophagous beetles [21,24,25,53], implying they have an important biological function, it is striking that so few hemicellulolytic enzymes (such as xylanases and mannanases) have emerged in the course of Phytophaga beetle evolution. The distribution of hemicellulolytic enzymes in coleopteran species may hint at either a strong variation in the abundance of hemicellulosic polysaccharides in different plant species or plant organs, or at more specific biological requirements for breaking down hemicellulose according to the insect. We would also like to point out that it is still unclear whether symbiotic microbes are involved in plant cell wall degradation in beetles; if the microbes are, they may potentially be providing hemicellulolytic enzymes in the gut of insects that lack endogenous enzymes. The ability of *C*. *maculatus* to break down GalM, GluM, CMC and xylan by expressing only GH5_10 proteins likely evolved through subfunctionalization events. In an evolutionary context, this ability seems to be a consequential event, as no cellulases or xylanases of any other GH family were found in the larval transcriptome of *C*. *maculatus* [21]. Altogether, and taking into account our previous work on the *C*. *maculatus* GH28 proteins [23], this beetle species possesses the ability to almost completely break down the polysaccharides of the plant’s primary cell wall by expressing only two GH families. CMA1, CMA4 and CMA5 (a likely allele of CMA4) exhibited no enzymatic activity against the substrates tested, although no substitution of important catalytic residues was observed. Interestingly, the expression of CMA4 transcripts in the gut tissue as well as the presence of the corresponding protein in the gut lumen of *C*. *maculatus* larvae have been reported elsewhere [45,46], suggesting that this protein plays an important role in the gut of *C*. *maculatus*. The inability to degrade any of the substrates we tested does not exclude the possibility that these proteins are still active enzymes for which no substrate has yet been found. This fact could also suggest a neo-functionalization of these proteins, but this possibility remains to be investigated.

To learn more about the physiological function of the *G*. *viridula* GH5_10 mannanase, we performed RNAi experiments. We successfully managed to knock down the expression of GVI1, which correlated with a reduction of enzymatic activity against galactomannan, indicating that GVI1 is the only mannanase expressed in the gut of *G*. *viridula*. However, we found no significant differences regarding either growth rate or mortality between GVI1-silenced larvae and the GFP control. These results would imply that GVI1 may not fulfill a primary digestive function, such as providing degraded plant cell wall polysaccharides (e.g. manno-oligomers or mannose) for metabolic purposes, e.g., glycolysis or fatty acid metabolism. We hypothesize instead that cleaving hemicellulosic components of the plant’s cell wall is rather accessory and may facilitate exposure of plant cells to the insect, allowing *G*. *viridula* to gain access to and to benefit from simple sugars and proteins present in plant cells. Although the previous hypothesis may also elicit reduced growth and/or the increased mortality of silenced larvae, the activity of GVI1 may be compensated for by other glycoside hydrolase families encoded by *G*.*viridula*, such as GH45 cellulases and GH28 pectinases. But such speculation needs to be further examined by, for example, performing comparative expression profiling using RNA-Seq between silenced and control insects. Interestingly, Nogueira *et al*. demonstrated that *C*. *maculatus* treated with a cysteine peptidase inhibitor responded by up-regulating other digestive enzymes, including CMA3 [46]. This finding may suggest a compensation of inhibited proteases by other digestive enzymes or it is a stress response due to the decreasing amount of nitrogen set free in the digestive tract of the animal. The former hypothesis seems rather unlikely, as mannan degradation does not directly increase nitrogen levels. Thus, we believe that a stress response is rather likely and this hypothesis has been already suggested elsewhere [54].

GH5 is a large multigene family, but its members are rarely found in Coleoptera or in insects in general; to date only three subfamilies (2, 8 and 10) have been identified [21,25–27,55]. Our investigation revealed that, according to data currently available, only 59 genes encoding GH5_10 are present within the tree of life, and, in fact, this gene family seems completely absent from plants and fungi. Of those 59 genes, only five are found in two species of Coleoptera. We can also exclude bacterial contamination because the GH5_10 genes of *G*. *viridula* and *C*. *maculatus* were found to harbor introns. Contamination by fungal-derived GH5s is also unlikely because, as previously mentioned, genes encoding GH5_10 seem absent in fungi. To our surprise, our phylogenetic analysis revealed that coleopteran-derived GH5_10 proteins did not cluster together with other insect counterparts but, rather, with those derived from three different species of mites belonging to the Oribatidae (Chelicerata). Additional GH5_10 sequences were identified in transcriptomes of insects belonging to orders other than Coleoptera, i.e. Zygentoma, Archaeognatha and Ephemeroptera. The latter sequences clustered together with collembolan- and crustacean-derived GH5_10 sequences. The patchy distribution of those proteins within arthropods indicates that the apparition of GH5_10 genes happened several times individually or else a massive gene loss has occurred in this phylum. As the latter hypothesis seems unlikely, we believe that the most parsimonious explanation for the appearance of GH5_10 genes in arthropods is the occurrence of several independent horizontal gene transfer (HGT) events, probably from bacteria to arthropod, occurring at several time points in the evolution of arthropods. However, we would like to note that because of the few bacteria-derived GH5_10 sequences currently available, our phylogenetic analysis has a poor resolution. Although we cannot fully support the hypothesis of an HGT event, the availability of an increasing amount of bacteria-derived GH5_10 sequences in the near future may solve this problem.

As we investigated the structure of the genes encoding GH5_10 in *G*. *viridula* and *C*. *maculatus*, we realized that the position and phase of the first and last introns are shared between these sequences. A logical explanation for this observation is that the most recent common ancestor of these two species of chrysomelid beetles possessed at least one GH5_10 gene harboring these two conserved introns. Subsequently, if the hypothesis for the acquisition of this gene family through an HGT event is true, this transfer should have happened at least in the most recent common ancestor of these two beetle species. Apart from *G*. *viridula* and *C*. *maculatus*, and according to the state of transcriptome and genome data currently available, genes encoding GH5_10 are apparently absent from other species of Chrysomelidae and even from species within the superfamily Chrysomeloidea and its sister superfamily Curculionoidea [21,25,28,53]. Altogether, it implies that a major gene loss happened among Chrysomelidae. An HGT event between these two species may also represent an alternative hypothesis, but this remains to be investigated.

Finally, the restriction of GH5_10 proteins to confined animal lineages may have represented an important factor to allow these animals to adapt to their food. Although the advantage of having mannanases is understandable for *C*. *maculatus* and other species of Bruchinae, it is less clear how *G*. *viridula* would benefit. In fact, species of Bruchinae often use the seeds of legumes as a food source. Legume seeds are notoriously rich in galactomannan, which is used as a storage sugar and subsequently as a source of energy during germination [7,56]. For an insect feeding on these seeds, the ability to break down galactomannan and use it as a potential source of energy represents a true advantage. We strongly believe that, within Bruchinae, the presence of GH5_10 is common and not limited to *C*. *maculatus*. A similar situation has been observed in the coffee berry borer, *H*. *hampei*—a species that feeds on coffee beans, which are also very rich in mannans [7] -which has acquired a GH5_8 mannanase from bacteria through HGT [27]. On the other hand, our RNAi experiments indicated that knocking down the expression of GVI1, which correlated with a drastic reduction of the mannanase activity in the gut of silenced larvae, neither significantly decreased growth nor increased mortality compared to control larvae. We also could not find any information in the literature which would indicate that the content of mannans in *Rumex* sp. leaves is unusually high. Yet when GVI1 was put in contact with a preparation of plant cell wall isolated from *Rumex* leaves, mannan oligomers were visible on TLC, indicating that this enzyme likely contributes to breaking down the plant cell wall when the beetle feeds on its host plant.

## Supporting information

S1 Fig Amino acid alignment of beetle-derived GH5_10 proteins with two others for which the crystal structure has been resolved. Amino acid sequences were aligned without their predicted amino-terminal signal peptide. Conserved sites are depicted from dark to light blue, depending on the degree of amino acid identity. The two catalytic glutamate residues are indicated in red. Active site residues are indicated by arrows. The two reference sequences for which the crystal structure has been resolved are derived from the Antarctic springtail, *cryptopygus antarcticus* (CAN1, PDB: 4OOU_A), and from the blue mussel, *Mytilus edulis* (MED1, PDB: 2C0H_A).
(TIF)

S2 Fig Tissue-specific expression of beetle-derived genes encoding GH5_10 proteins. Late-instar actively feeding larvae were dissected, and gut and rest bodies were used for total RNA preparation and quantitative RT-PCR. (A) The gene encoding GVI1 is significantly more expressed in the gut of *G*. *viridula* larvae compared to in the rest of the body. The gene expression is given as the copy number of GVI1 per 1000 molecules of RPS3 (control gene) ± SEM. (B) Genes encoding GH5_10 are significantly more expressed in the gut of *C*. *maculatus* larvae compared to in the rest of the body. The gene expression is given as the copy number of GVI1 per 1000 molecules of EF1α (control gene) ± SEM. Data were plotted using a log-transformed scale. Gene expression data were analyzed using paired t-tests (statistical values see S3 Table).
(TIF)

S3 Fig Zymogram of the mannanase activity after RNAi in *G*. *viridula*. The same protein samples (day 4 post injection) as those described in Fig 4 were used for zymographic analyses. (A) 5 μg total proteins were loaded on a semi-native SDS-PAGE gel containing 0.1% (w/v) galactomannan. After the run, the gel was stained with Coomassie and used as a loading control. (B) 0.5 μg total proteins from the same samples were loaded on the same semi-native SDS-PAGE gel. After the run, this part of the gel was used to detect mannanase activity and activity bands were detected after staining with Congo red. iGH5: samples were prepared from *G*. *viridula* larvae injected with dsRNA targeting GVI1; iGFP: samples were prepared from larvae injected with dsRNA targeting GFP and used as controls; NIC: non-injected control larvae.
(TIF)

S4 Fig Action of GVI1 on a preparation of plant cell wall from *Rumex obtusifolius* leaves. GVI1 was heterologously expressed in *Sf*9 cells and crude enzyme extract was incubated with a preparation of protein-free plant cell wall (PCW) isolated from *R*. *obtusifolius* leaves. Results were analyzed on TLC. A reaction in which GVI1 had been omitted was included as a control. In addition, the PCW was also incubated with a commercially available control cellulase preparation (CCP) isolated from *Trichoderma reesei*. Several standards were used: from mannose (M1) to mannohexaose (M6); from glucose (G1) to cellopentaose (G5); from xylose (X1) to xylotriose (X3).
(TIF)

S5 Fig Conservation of intron position and phase between *G*. *viridula* and *C*. *maculatus* GH5_10 genes. The amino acid sequences of *G*. *viridula* GVI1 and *C*. *maculatus* CMA1 to CMA4 were aligned using MUSCLE. The sequence corresponding to the signal peptide is indicated in bold. The *G*. *viridula* GVI1 gene was amplified by PCR using gDNA as a template. The sequences corresponding to the *C*. *maculatus* GH5_10 genes were retrieved from a genome draft assembly of this species (http://www.beanbeetles.org/genome/). Missing sequence data for the *C*. *maculatus* GH5_10 genes are indicated in gray. Intron positions and phase are indicated by colored amino acids. Amino acids in green correspond to the insertion of a phase 0 intron. Amino acids in red correspond to the insertion of a phase 1 intron. Amino acids in blue correspond to the insertion of a phase 2 intron.
(TIF)

S1 Table List of primers used in this study. (PDF)

S2 Table Details of the amino acid sequences used for the phylogenetic analysis. (PDF)

S3 Table Statistical analysis of tissue specific gene expression (S2 Fig). (PDF)
