# Supplementary figures and images for: Evolution and functional characterization of CAZymes belonging to subfamily 10 of glycoside hydrolase family 5 (GH5_10) in two species of phytophagous beetles
Source: PLoS One. 2017 Aug 30;12(8):e0184305. doi: 10.1371/journal.pone.0184305 (PMC5576741; doi:10.1371/journal.pone.0184305)

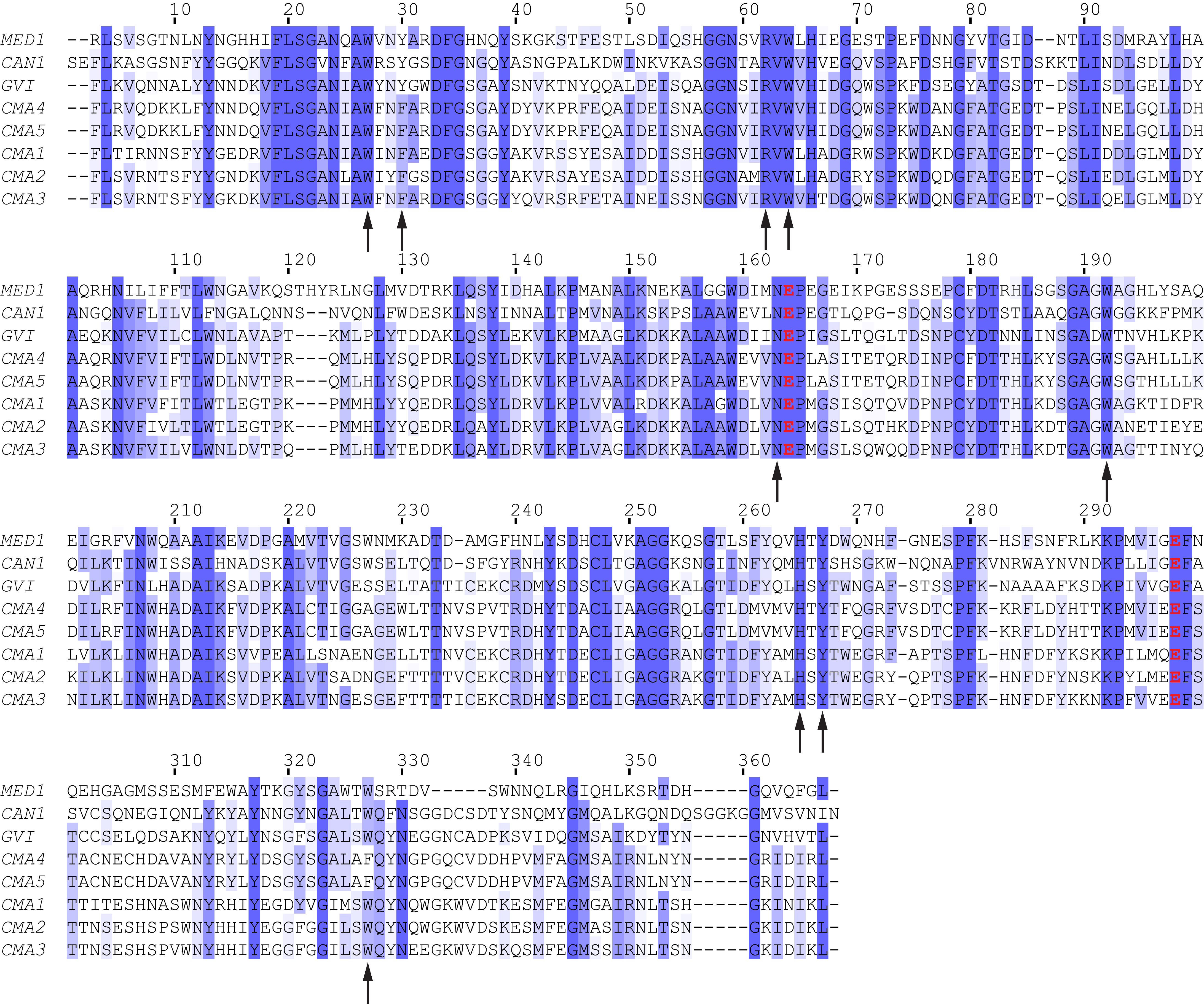

Supplement: S1 Fig — Amino acid sequences were aligned without their predicted amino-terminal signal peptide. Conserved sites are depicted from dark to light blue, depending on the degree of amino acid identity. The two catalytic glutamate residues are indicated in red. Active site residues are indicated by arrows. The two reference sequences for which the crystal structure has been resolved are derived from the Antarctic springtail, cryptopygus antarcticus (CAN1, PDB: 4OOU_A), and from the blue mussel, Mytilus edulis (MED1, PDB: 2C0H_A). (TIF) [file pone.0184305.s001.tif]

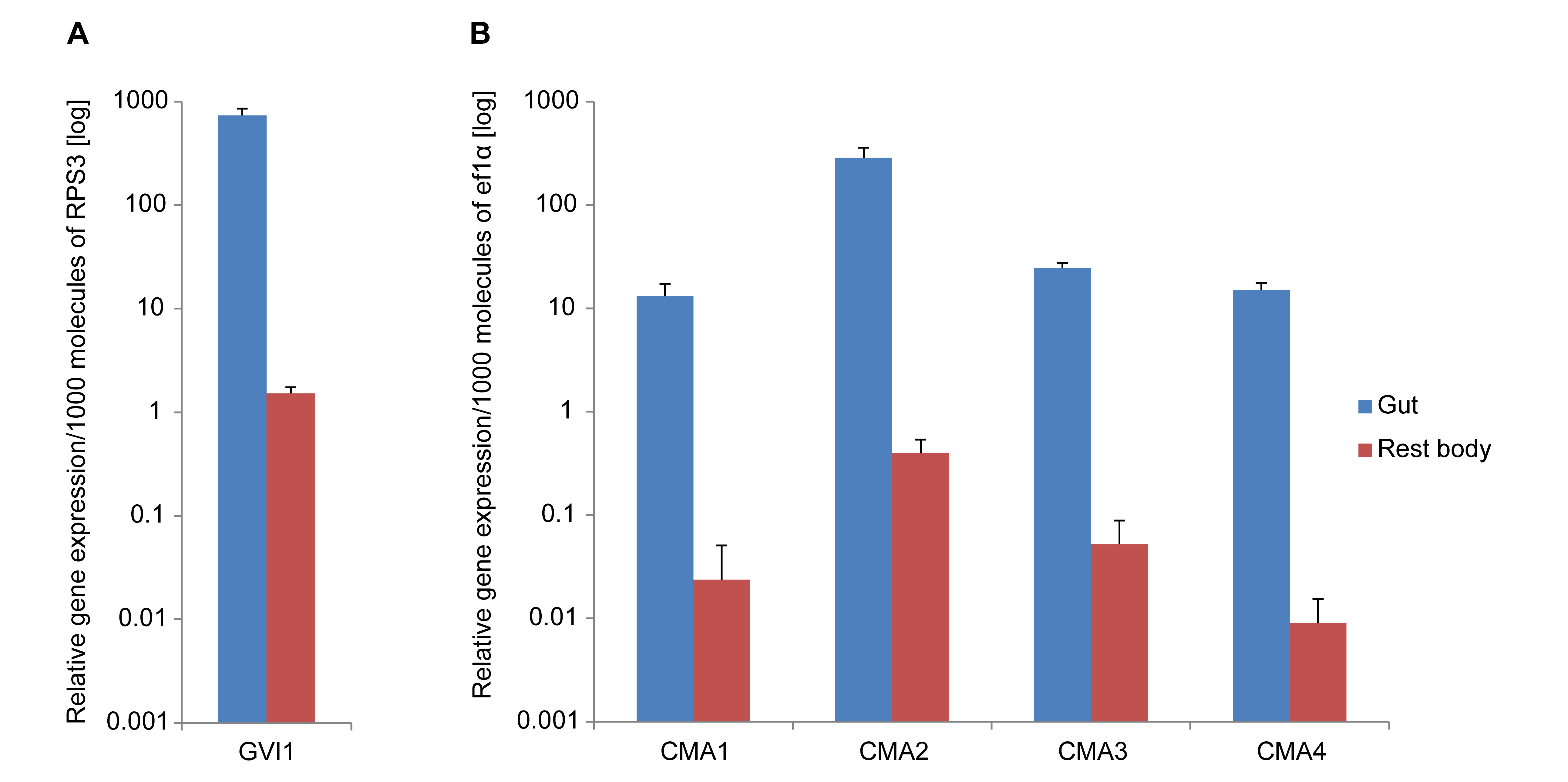

Supplement: S2 Fig — Late-instar actively feeding larvae were dissected, and gut and rest bodies were used for total RNA preparation and quantitative RT-PCR. (A) The gene encoding GVI1 is significantly more expressed in the gut of G. viridula larvae compared to in the rest of the body. The gene expression is given as the copy number of GVI1 per 1000 molecules of RPS3 (control gene) ± SEM. (B) Genes encoding GH5_10 are significantly more expressed in the gut of C. maculatus larvae compared to in the rest of the body. The gene expression is given as the copy number of GVI1 per 1000 molecules of EF1α (control gene) ± SEM. Data were plotted using a log-transformed scale. Gene expression data were analyzed using paired t-tests (statistical values see S3 Table). (TIF) [file pone.0184305.s002.tif]

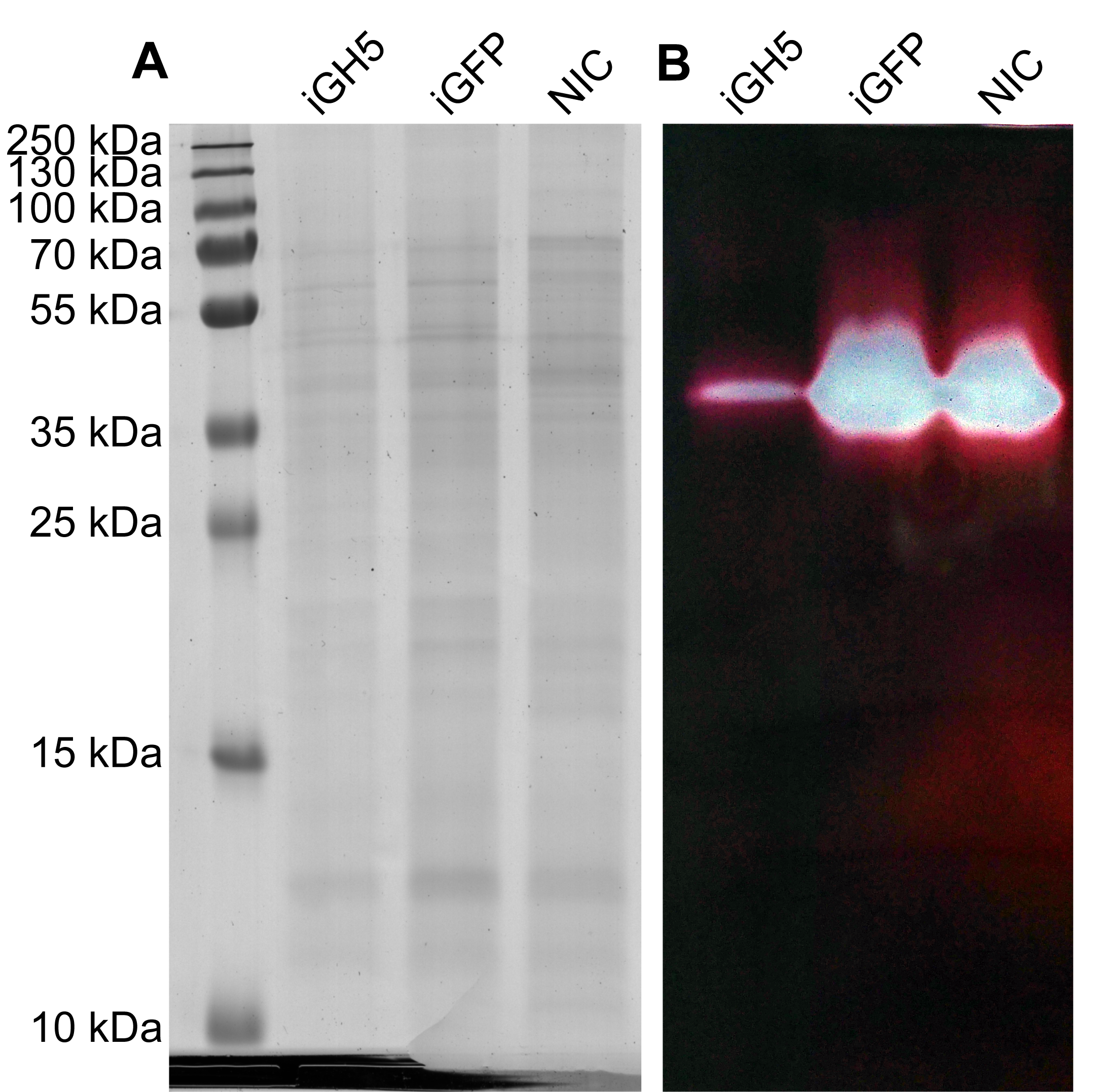

Supplement: S3 Fig — The same protein samples (day 4 post injection) as those described in Fig 4 were used for zymographic analyses. (A) 5 μg total proteins were loaded on a semi-native SDS-PAGE gel containing 0.1% (w/v) galactomannan. After the run, the gel was stained with Coomassie and used as a loading control. (B) 0.5 μg total proteins from the same samples were loaded on the same semi-native SDS-PAGE gel. After the run, this part of the gel was used to detect mannanase activity and activity bands were detected after staining with Congo red. iGH5: samples were prepared from G. viridula larvae injected with dsRNA targeting GVI1; iGFP: samples were prepared from larvae injected with dsRNA targeting GFP and used as controls; NIC: non-injected control larvae. (TIF) [file pone.0184305.s003.tif]

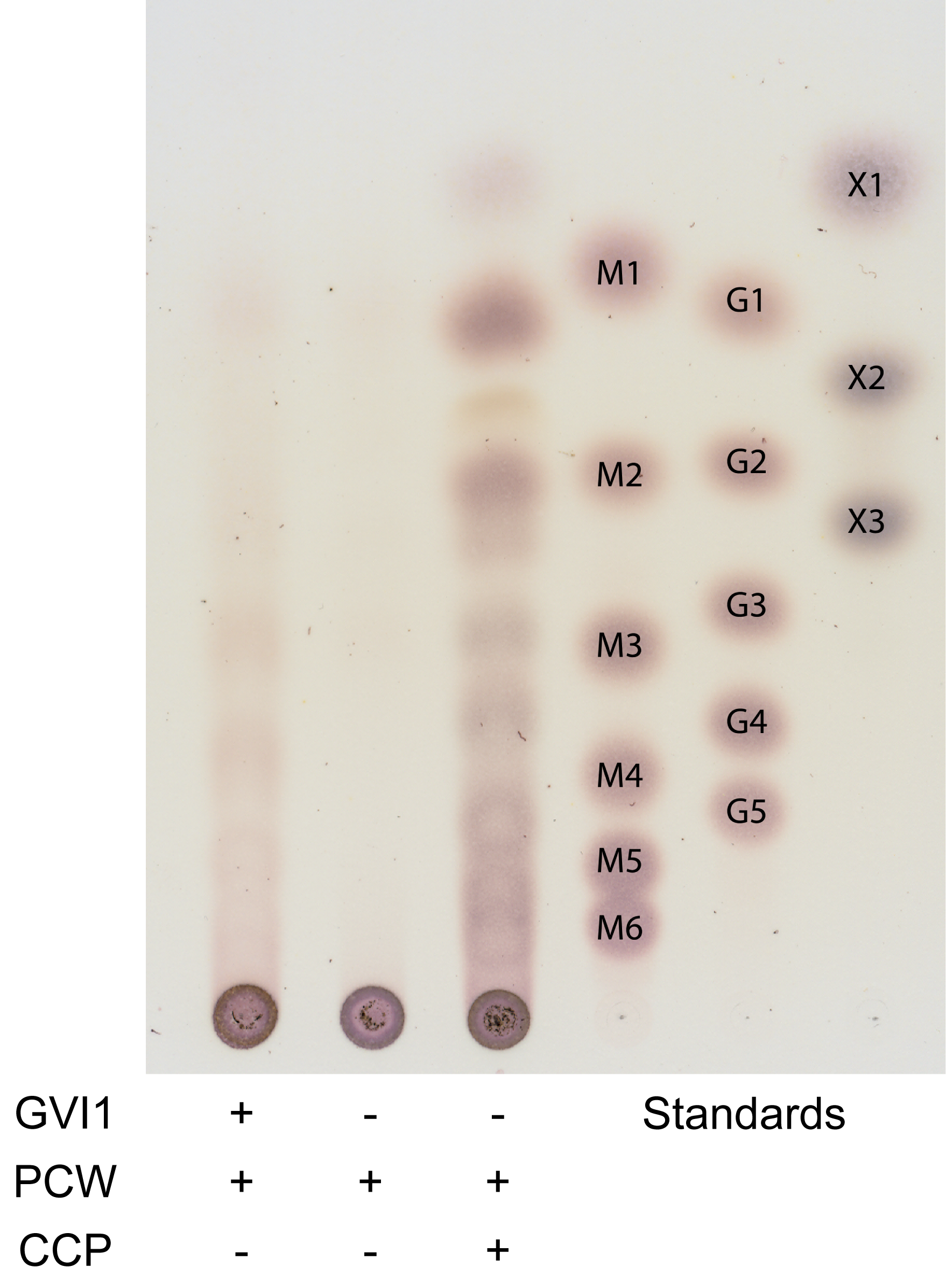

Supplement: S4 Fig — GVI1 was heterologously expressed in Sf9 cells and crude enzyme extract was incubated with a preparation of protein-free plant cell wall (PCW) isolated from R. obtusifolius leaves. Results were analyzed on TLC. A reaction in which GVI1 had been omitted was included as a control. In addition, the PCW was also incubated with a commercially available control cellulase preparation (CCP) isolated from Trichoderma reesei. Several standards were used: from mannose (M1) to mannohexaose (M6); from glucose (G1) to cellopentaose (G5); from xylose (X1) to xylotriose (X3). (TIF) [file pone.0184305.s004.tif]

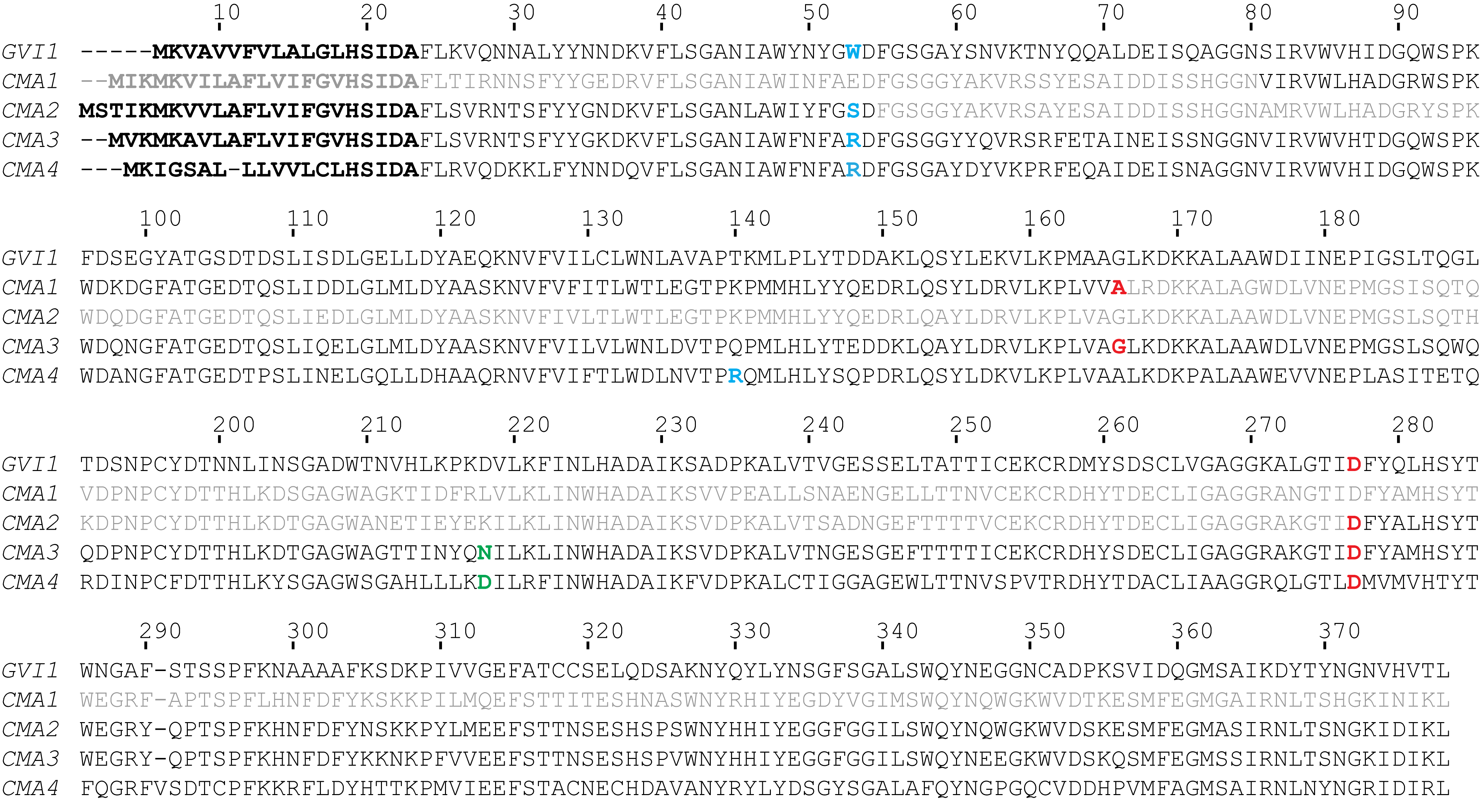

Supplement: S5 Fig — The amino acid sequences of G. viridula GVI1 and C. maculatus CMA1 to CMA4 were aligned using MUSCLE. The sequence corresponding to the signal peptide is indicated in bold. The G. viridula GVI1 gene was amplified by PCR using gDNA as a template. The sequences corresponding to the C. maculatus GH5_10 genes were retrieved from a genome draft assembly of this species (http://www.beanbeetles.org/genome/). Missing sequence data for the C. maculatus GH5_10 genes are indicated in gray. Intron positions and phase are indicated by colored amino acids. Amino acids in green correspond to the insertion of a phase 0 intron. Amino acids in red correspond to the insertion of a phase 1 intron. Amino acids in blue correspond to the insertion of a phase 2 intron. (TIF) [file pone.0184305.s005.tif]
