# Supplementary material for: Evolution and functional characterization of CAZymes belonging to subfamily 10 of glycoside hydrolase family 5 (GH5_10) in two species of phytophagous beetles
Source: PLoS One. 2017 Aug 30;12(8):e0184305. doi: 10.1371/journal.pone.0184305 (PMC5576741; doi:10.1371/journal.pone.0184305)
Supplement: S1 Table — (PDF) [file pone.0184305.s006.pdf]

S1 Table. List of primers used in this study.

Gene	Primer name	Sequence (5' – 3')	Function
GVI1	GVI1_ORF_F	ACCATGGAAGTCGCTGTGGTATTCG	Cloning pIB/V5-His TOPO/TA
GVI1	GVI1_ORF_R	AAGGGTCACATGGACATTGCCGTTG	Cloning pIB/V5-His TOPO/TA
CMA1	CMA1_ORF_F	ACCATGGTCAAGATGAAGGTGAT	Cloning pIB/V5-His TOPO/TA
CMA1	CMA1_ORF_R	CAACTTTATATTAATCTTTCCATGG	Cloning pIB/V5-His TOPO/TA
CMA2	CMA2_ORF_F	ACCATGGCCACGATCAAGATGAAGGT	Cloning pIB/V5-His TOPO/TA
CMA2	CMA2_ORF_R	CAACTTTATATCAATCTTTCCGTTG	Cloning pIB/V5-His TOPO/TA
CMA3	CMA3_ORF_F	ACCATGGTCAAGATGAAAGCGGT	Cloning pIB/V5-His TOPO/TA
CMA3	CMA3_ORF_R	CAACTTTATGTCAATCTTTCCATTG	Cloning pIB/V5-His TOPO/TA
CMA4	CMA4_ORF_F	ACCATGGAAATTGGATCCGCTCTGCT	Cloning pIB/V5-His TOPO/TA
CMA4	CMA4_ORF_R	CAGCCGAATATCAATCCTCCCA	Cloning pIB/V5-His TOPO/TA
CMA5	CMA5_ORF_F	ACCATGGAACTTGGATCCGCACTGCT	Cloning pIB/V5-His TOPO/TA
CMA5	CMA5_ORF_R	CAGCCTAATATCAATCCTCCCA	Cloning pIB/V5-His TOPO/TA
GVI1	GVI1_dsRNA_F	TAATACGACTCACTATAGGGAGCACCAACAACTTGATCAACAG	dsRNA generation
GVI1	GVI1_dsRNA_R	TAATACGACTCACTATAGGGAGTTCTTGAAGGGACTGGAGGT	dsRNA generation
GFP	GFP_dsRNA_F	TAATACGACTCACTATAGGCACATGAAGCAGCACGACTT	dsRNA generation
GFP	GFP_dsRNA_R	TAATACGACTCACTATAGGTGCTCAGGTAGTGGTTGTCG	dsRNA generation
GVI1	GVI1_F	CAACTATGGATGGGACTTCG	Quantitative PCR
GVI1	GVI1_R	GATGTGCACCCAAACTCTGA	Quantitative PCR
GVI_RpS3	GVI_RpS3_F	GCAGGATCCGTGAGTTGAC	Quantitative PCR
GVI_RpS3	GVI_RpS3_R	ACAGGCCTCGATTGGCTAC	Quantitative PCR
CMA1	CMA1_F	AGAAGACACCCAATCGCTGA	Quantitative PCR
CMA1	CMA1_R	CTGCAGCCTATCTTCCTGGT	Quantitative PCR
CMA2	CMA2_F	ACTCTGTGGACTTTGGAGGG	Quantitative PCR
CMA2	CMA2_R	TTGTCTTTCAGGCCTGCAAC	Quantitative PCR
CMA3	CMA3_F	TCAGGAGAGTTCACCACCAC	Quantitative PCR
CMA3	CMA3_R	CTGTGCATGGCGTAGAAGTC	Quantitative PCR
CMA4	CMA4_F	AACCCTTGCTTTGACACCAC	Quantitative PCR
CMA4	CMA4_R	CACAGCGCTTTAGGATCCAC	Quantitative PCR
CMA_EF1α	CMA_EF1α_F	AAGGCCTCCACACACATAGG	Quantitative PCR
CMA_EF1α	CMA_EF1α_R	AAGGTTGATCGTCGTTCTGG	Quantitative PCR
