# Supplementary material for: Evolution and functional characterization of CAZymes belonging to subfamily 10 of glycoside hydrolase family 5 (GH5_10) in two species of phytophagous beetles
Source: PLoS One. 2017 Aug 30;12(8):e0184305. doi: 10.1371/journal.pone.0184305 (PMC5576741; doi:10.1371/journal.pone.0184305)
Supplement: S2 Table — (PDF) [file pone.0184305.s007.pdf]

S2_Table. Details of the amino acid sequences used for the phylogenetic analysis.

Specie name	abbreviation	Accession number	Notes
**Bacteria**			
Fibrobacter succinogenes subsp. succinogenes S85	FSU1	ACX74338.1	Fibrobacteres
Flammeovirga sp. MY04	FLA1	ANQ49303.1	Bacteroidetes
Flammeovirga sp. MY04	FLA2	ANQ52741.1	Bacteroidetes
Flammeovirga yaeyamensis	FYA1	ACA05117.2	Bacteroidetes
Melioribacter roseus P3M-2	MRO1	AFN74927.1	Ignavibacteriae
Saccharophagus degradans 2-40	SDE1	ABD81545.1	Proteobacteria (gamma)
Spirochaeta thermophila DSM 6192	STH1	ADN01068.1	Spirochaetes
Spirochaeta thermophila DSM 6578	STH2	AEJ60366.1	Spirochaetes
Uncultured bacterium	UBA1	AEV59732.1	
Vibrio natriegens	VNA1	ANQ24656.1	Proteobacteria (gamma)
**Insects**			
Gastrophysa viridula	GVI1	ADU33333.1	Arthropoda (Coleoptera)
Callosobruchus maculatus	CMA1	ADU33271.1	Arthropoda (Coleoptera)
Callosobruchus maculatus	CMA2	ADU33272.1	Arthropoda (Coleoptera)
Callosobruchus maculatus	CMA3	ADU33273.1	Arthropoda (Coleoptera)
Callosobruchus maculatus	CMA4	ADU33274.1	Arthropoda (Coleoptera)
Tricholepidion gertschi	TGE1	GASO02036642	Arthropoda (Zygentoma)
Tricholepidion gertschi	TGE2	GASO02042472	Arthropoda (Zygentoma)
Thermobia domestica	TDO1	GASN02045897	Arthropoda (Zygentoma)
Thermobia domestica	TDO2	GASN02045380	Arthropoda (Zygentoma)
Machilis hrabei	MHR1	GAUM02040243	Arthropoda (Archaeognatha)
Eurylophella sp. AD-2013	EUR1	GAZG02013172	Arthropoda (Ephemeroptera)
Isonychia bicolor	IBI1	GAXA02038640	Arthropoda (Ephemeroptera)
**Collembola**			
Cryptopygus antarcticus	CAN1	ABV68808.1	Arthropoda
Orchesella cincta OC12957	OCI1	GAMM01012947	Arthropoda
Sminthurus viridis	SVI1	GATZ02024785	Arthropoda
Folsomia candida	FCA1	GASX02002882	Arthropoda
Pogonognathellus sp. AD-2013	POG1	GATD02008031	Arthropoda
Tetrodontophora bielanensis	TBI1	GAXI02022226	Arthropoda
**Crustacea**			
Limnoria quadripunctata	LQU1	ADE58567.1	Arthropoda
Limnoria quadripunctata	LQU2	ADE58568.1	Arthropoda
Limnoria quadripunctata	LQU3	ADE58569.1	Arthropoda
Hyalella azteca	HAZ1	XP_018022370.1	Arthropoda
Daphnia pulex	DPU1	EFX71596.1	Arthropoda
Daphnia pulex	DPU2	EFX71597.1	Arthropoda
Proasellus grafi	PGR1	HAEX01030896	Arthropoda
Talitrus saltator	TSA1	GDUJ01040948	Arthropoda
Cherax quadricarinatus	CQU1	HACK01027957	Arthropoda
**Chelicerata**			
Nothrus palustris	NPA1	GEYJ01076133	Arthropoda
Nothrus palustris	NPA2	GEYJ01112851	Arthropoda
Nothrus palustris	NPA3	GEYJ01073735	Arthropoda
Platynothrus peltifer	PPE1	GEYZ01027687	Arthropoda
Platynothrus peltifer	PPE2	GEYZ01016327	Arthropoda
Platynothrus peltifer	PPE3	GEYZ01010130	Arthropoda
Steganacarus magnus	SMA1	GEYQ01012964	Arthropoda
Steganacarus magnus	SMA2	GEYQ01047325	Arthropoda
**Gastropoda**			
Aplysia kurodai	AKU1	BAJ60954.1	Mollusca
Biomphalaria glabrata	BGL1	AAV91523.1	Mollusca
Haliotis discus discus	HDI1	ACJ12612.1	Mollusca
Haliotis discus discus	HDI2	ACJ12613.1	Mollusca
Haliotis discus hannai	HDI3	BAE78456.1	Mollusca
Limacina antarctica	LAN1	GDRM01030628	Mollusca
Elysia timida	ETI1	GBRM01102742	Mollusca
Deroceras reticulatum	DRE1	JW037389	Mollusca
Pomacea canaliculata	PCA1	GBZZ01045211	Mollusca

**Bivalvia**			
Mytilus edulis	MED1	CAC81056.1	Mollusca
Scapharca broughtonii	SBR1	GEXI01012405	Mollusca
Crassostrea gigas	CGI1	GECI01038763	Mollusca
Villosa lienosa	VLI1	JR521835	Mollusca
