# Supplementary material for: Evolution and functional characterization of CAZymes belonging to subfamily 10 of glycoside hydrolase family 5 (GH5_10) in two species of phytophagous beetles
Source: PLoS One. 2017 Aug 30;12(8):e0184305. doi: 10.1371/journal.pone.0184305 (PMC5576741; doi:10.1371/journal.pone.0184305)
Supplement: S3 Table — (PDF) [file pone.0184305.s008.pdf]

S3 Table. Statistical analysis of tissue specific gene expression (Fig S2).

Species	Gene	t-value	p-value
G.viridula	GH5	16.092	0.004
C. maculatus	GH5-1	4.525	0.006
	GH5-2	9.019	0.003
	GH5-3	30.468	<0.001
	GH5-4	7.422	0.002
